# Downscaling of far-red solar-induced chlorophyll fluorescence of different crops from canopy to leaf level using a diurnal data set acquired by the airborne imaging spectrometer HyPlant

**DOI:** 10.1016/j.rse.2021.112609

**Published:** 2021-10

**Authors:** Bastian Siegmann, Maria Pilar Cendrero-Mateo, Sergio Cogliati, Alexander Damm, John Gamon, David Herrera, Christoph Jedmowski, Laura Verena Junker-Frohn, Thorsten Kraska, Onno Muller, Patrick Rademske, Christiaan van der Tol, Juan Quiros-Vargas, Peiqi Yang, Uwe Rascher

**Affiliations:** a Institute of Bio- and Geosciences, IBG-2: Plant Sciences, Forschungszentrum Jülich GmbH, Wilhelm-Johnen-Straße, 52428 Jülich, Germany; b Laboratory of Earth Observation, Image Processing Laboratory, University of Valencia, C/ Catedrático José Beltrán, 2, 46980 Paterna, Valencia, Spain; c Remote Sensing of Environmental Dynamics Lab., DISAT, University of Milano-Bicocca, Piazza della Scienza 1, 20126 Milano, Italy; d Department of Geography, University of Zurich, Winterthurerstrasse 190, 8057 Zurich, Switzerland; e Department of Earth and Atmospheric Sciences and Department of Biological Sciences, University of Alberta, 11335 Saskatchewan Drive, Edmonton, AB T6G 2E3, Canada; f Center for Advanced Land Management Information Technologies, School of Natural Resources, University of Nebraska–Lincoln, 3310 Holdrege Street, Lincoln, NE 68583, USA; g Field Lab Campus Klein-Altendorf, Faculty of Agriculture, University of Bonn, Campus Klein-Altendorf 1, 53359 Rheinbach, Germany; h Faculty of Geo-Information Science and Earth Observation (ITC), University of Twente, Hengelosestraat 99, Enschede, 7500, AE, the Netherlands

**Keywords:** Solar-induced chlorophyll fluorescence, SIF, HyPlant, Diurnal course, Fluorescence correction vegetation index, FCVI, Fluorescence escape fraction, Photosynthetically active radiation

## Abstract

Remote sensing-based measurements of solar-induced chlorophyll fluorescence (SIF) are useful for assessing plant functioning at different spatial and temporal scales. SIF is the most direct measure of photosynthesis and is therefore considered important to advance capacity for the monitoring of gross primary production (GPP) while it has also been suggested that its yield facilitates the early detection of vegetation stress. However, due to the influence of different confounding effects, the apparent SIF signal measured at canopy level differs from the fluorescence emitted at leaf level, which makes its physiological interpretation challenging. One of these effects is the scattering of SIF emitted from leaves on its way through the canopy. The escape fraction ($f_{\mathrm{esc}}$) describes the scattering of SIF within the canopy and corresponds to the ratio of apparent SIF at canopy level to SIF at leaf level. In the present study, the fluorescence correction vegetation index (FCVI) was used to determine $f_{\mathrm{esc}}$ of far-red SIF for three structurally different crops (sugar beet, winter wheat, and fruit trees) from a diurnal data set recorded by the airborne imaging spectrometer HyPlant. This unique data set, for the first time, allowed a joint analysis of spatial and temporal dynamics of structural effects and thus the downscaling of far-red SIF from canopy (${\mathrm{SIF}}_{760}^{\mathrm{canopy}}$) to leaf level (${\mathrm{SIF}}_{760}^{\mathrm{leaf}}$). For a homogeneous crop such as winter wheat, it seems to be sufficient to determine $f_{\mathrm{esc}}$ once a day to reliably scale SIF_760_ from canopy to leaf level. In contrast, for more complex canopies such as fruit trees, calculating $f_{\mathrm{esc}}$ for each observation time throughout the day is strongly recommended. The compensation for structural effects, in combination with normalizing SIF_760_ to remove the effect of incoming radiation, further allowed the estimation of SIF emission efficiency ($\varepsilon_{\mathrm{SIF}}$) at leaf level, a parameter directly related to the diurnal variations of plant photosynthetic efficiency.

## Highlights

• The far-red SIF (SIF_760_) escape fraction (f_esc_) strongly depends on the crop type
• Crops with complex canopy geometries show more spatio-temporal variability in f_esc_
• Detection of diurnal variations in plant physiology from airborne SIF_760_ maps

## 1 Introduction

Spatially resolved information on the status of plants is vital in ecosystem research to gain a better understanding of plant functioning and productivity. Remote sensing data recorded from satellites and aircraft have provided such information for decades. Most of the approaches for monitoring vegetation conditions, however, were based solely on estimates of vegetation greenness derived from vegetation indices (VIs), which only allow observations of changes in potential photosynthesis (Campbell et al., 2019; Rossini et al., 2015). In contrast, solar-induced chlorophyll fluorescence (SIF) is the most direct measure of photosynthetic activity (ESA, 2015), since it is emitted from the core of the photosynthetic machinery (Meroni et al., 2009; Porcar-Castell et al., 2014). Although additional complications in interpreting SIF arise from the confounding effect of non-photochemical quenching (NPQ), SIF is sensitive to track dynamic changes in photosynthetic activity. This determines the importance of SIF in ecosystem research, e.g., for the monitoring of gross primary productivity (GPP) and the early detection of vegetation stress before it becomes detectable with conventional greenness-based remote sensing proxies (Ač et al., 2015; Campbell et al., 2008; Cheng et al., 2013).

The SIF signal is a continuous emission spectrum in the range of red and far-red light (650–850 nm) immediately released from chloroplasts after the absorption of sun light. It is related to photosynthesis and competes for absorbed excitation energy (PAR) with photochemistry (photochemical quenching, PQ) and thermal energy dissipation (NPQ) (Campbell et al., 2019; Magney et al., 2020, Mohammed et al., 2019; Porcar-Castell et al., 2014). Although active fluorescence techniques have been established as a means to assess leaf-level photosynthesis for decades (Murchie et al., 2018), the use of the SIF signal is relatively recent and many details of its application remain unclear.

The detection of SIF is challenging because it is only a small part of the reflected radiance (1–5%) measured by remote sensing instruments. In the last decade, however, several studies have demonstrated the capabilities of proximal (Jiang et al., 2020; Pinto et al., 2016), airborne (Damm et al., 2014; Siegmann et al., 2019), and satellite imaging sensors (Köhler et al., 2018; Sun et al., 2018) measuring SIF at different spatial scales and different temporal resolutions.

The increasing availability of diurnal and seasonal time series of canopy SIF measured from remote sensing instruments is very useful in gaining a better understanding of what drives the SIF signal at different spatial and temporal scales. While diurnal SIF data are helpful in detecting plant stress caused by harsh environmental conditions, data from seasonal SIF time series can facilitate advanced crop yield estimates and the prediction of biomass accumulation. Recent studies have already demonstrated the potential of such data sets measured with point spectrometers (Wieneke et al., 2018) or proximal imaging devices (Pinto et al., 2016) to detect diurnal and seasonal dynamics in vegetation photosynthesis.

Several confounding effects challenge the correct physiological interpretation of retrieved canopy SIF. Accurate knowledge of these effects is required, including i) absorbed photosynthetically active radiation (APAR), ii) the involved complementary radiation pathways, i.e., NPQ, iii) scattering and reabsorption of SIF in the canopy, iv) scattering and absorption of SIF in the atmosphere and v) sensor effects on retrieved SIF (van der Tol et al., 2019; Cogliati et al., 2015; Porcar-Castell et al., 2014; Damm et al., 2011).

In particular, an understanding of re-absorption and scattering of SIF within the canopy is essential to comparing SIF observations from different scales and has therefore been a topic of great research interest in recent years. Both processes are wavelength-dependent and affected by canopy structure, e.g., leaf area, leaf orientation, and leaf clumping (Yang and van der Tol, 2018; Verrelst et al., 2015). While red SIF derived at the O_2_—B absorption feature at 687 nm (SIF_687_) has a higher probability of being re-absorbed, far-red SIF derived at the O_2_-A absorption feature at 760 nm (SIF_760_) is much more scattered (Porcar-Castell et al., 2014). Therefore, SIF measured at the canopy is different from SIF measured at the leaf scale and cannot be directly used to quantitatively detect variations in plant physiology (Migliavacca et al., 2017; van der Tol et al., 2016).

The SIF escape fraction ($f_{\mathrm{esc}}$) is calculated as the ratio of SIF at canopy level to SIF at leaf level and describes the scattering of SIF in the viewing direction (0 ≤$\;f_{\mathrm{esc}}\;$≤ 1) (Yang et al., 2020; Guanter et al., 2014). $f_{\mathrm{esc}}$ is determined by directly comparing SIF measured at leaf and canopy level. Recent examples of this kind of study include Romero et al. (2020) for pea, rye grass, and maize, Cendrero-Mateo et al. (2015) for wheat, and Fournier et al. (2012) for grassland. Since the experimental determination of scattering effects in the canopy is very labor-intensive and only representative for specific illumination conditions, viewing angles, and leaf properties (Cendrero-Mateo et al., 2015), Yang and van der Tol (2018) developed a more generalized method to correct the SIF_760_ emission of dense canopies for scattering effects. Their approach utilizes the similarity of the radiative transfer of intercepted incident light and emitted SIF_760_, which allows the calculation of SIF_760_ scattering as the ratio of near infrared top-of-canopy (TOC) reflectance ($R_{\mathrm{NIR}}$) to canopy interception ($\;i_{0}$) ($\;f_{\mathrm{esc}}$=$R_{\mathrm{NIR}}$/$i_{0}$). Using this relationship, Liu et al. (2019) estimated $\;f_{\mathrm{esc}}$ from TOC reflectance data based on random forest regression and were able to scale canopy SIF derived from non-imaging in situ measurements and imaging airborne measurements down to leaf level. Zeng et al. (2019) further exploited the relationship between $\;f_{\mathrm{esc}}$ and TOC near-infrared (NIR) reflectance and developed the near-infrared reflectance of vegetation (NIRv) index. To calculate NIRv, they multiplied NIR reflectance by the normalized difference vegetation index (NDVI) to account for soil effects and used the fraction of absorbed PAR (fAPAR) as a proxy of canopy interception. Thus, they ensured that the approach additionally was also usable to compute$\;f_{\mathrm{esc}}$ of sparse vegetation canopies$(f_{\mathrm{esc}}=R_{\mathrm{NIR}}\times \mathrm{NDVI}/\mathrm{fAPAR}$). Zhang et al. (2020) further modified this approach to determine $f_{\mathrm{esc}}$ to downscale far-red SIF derived from OCO-2 satellite data. In contrast to Zeng et al. (2019), the authors calculated canopy interception based on leaf area (LAI) and clumping index (CI) information derived from MODIS satellite data using an approach developed by Chen and Leblanc (2001). One drawback of the NIRv-based approach to determine f_esc_, however, is that it is not universally applicable, since some steps in the estimation of $f_{\mathrm{esc}}$ are not fully consistent with radiative transfer theory (Yang et al., 2020). To address this issue, Yang et al. (2020) introduced the fluorescence correction vegetation index (FCVI), which can be calculated as the difference of NIR reflectance at 770 nm and the averaged reflectance of the visible spectral range ($\mathrm{FCVI}=R_{\mathrm{NIR}}-R_{\bar{\mathrm{VIS}}}$). The FCVI is a surrogate of the product of $f_{\mathrm{esc}}$ and fAPAR of SIF_760_. The authors analytically demonstrated the relationship between both factors and TOC reflectance, and thus the consistency between the FCVI and the spectral invariant radiative transfer theory (Yang et al., 2020; Yang and van der Tol, 2018). Calculating the FCVI from TOC reflectance data with knowledge of fAPAR therefore allows the determination of $f_{\mathrm{esc}}$ of SIF_760_ ($f_{\mathrm{esc}}$=FCVI/fAPAR). However, compared to NIRv, the FCVI also has a number of drawbacks and is therefore not universally applicable. For instance, the FCVI is not suited to very sparse vegetation canopies and it also requires hyperspectral data in the visible spectral range for its calculation.

As a parameter representing the ability of plant canopies to absorb incident PAR, fAPAR is closely linked to parameters describing the structure and architecture of canopies. To this end, the plant type, leaf angle distribution (LAD), LAI, and leaf clumping are assumed to be the most relevant factors determining fAPAR (Asrar et al., 1984; Daughtry et al., 1992; Rahman et al., 2014). fAPAR can be measured directly using ground measurements, but also through the use of remote sensing techniques and VIs. However, the relationship is not universal and can vary for different sites, vegetation types, phenological stages, differences in soil fractions, and climatic zones (Tan et al., 2018; Roujean and Breon, 1995). A number of VIs have been tested for fAPAR prediction on different crops, and empirical correlations with the NDVI or simple ratio (SR) have been developed (Tan et al., 2013; Viña and Gitelson, 2005). In this study, the wide dynamic range vegetation index (WDRVI) (Gitelson, 2004), which is a modified version of the NDVI, was used to approximate fAPAR.

Existing studies addressing the scaling of SIF have only focused on either temporal dynamics for single point measurements (Yang et al., 2020) or on spatial dynamics for a single snapshot in time (Liu et al., 2019). Despite the highly interesting insights provided by previous studies, several research questions remain unsolved. These include the extent to which SIF_760_ retrieved at canopy level is influenced by the canopy structure across crop types and whether $\;f_{\mathrm{esc}}$ varies with illumination conditions over the course of a day. Furthermore, the reliability of the FCVI as a universal approach for scaling SIF_760_ from canopy to leaf level remains to be evaluated. Moreover, a better understanding is needed of the spatial and diurnal dynamics of SIF_760_ at leaf level as an important indicator of functional (physiological) vegetation responses to changing conditions.

Consequently, we hypothesized that a synergistic perspective on both temporal and spatial dynamics in SIF_760_ will complement existing insights and help to overcome as-yet unsolved scaling problems. The experimental data used in this study allow, for the first time, an investigation of the spatial and temporal dynamics of the canopy and leaf SIF_760_ of different crops. A diurnal data set recorded by the airborne imaging spectrometer HyPlant (Rascher et al., 2015) was used in combination with the aforementioned approach to derive $f_{\mathrm{esc}}$ of three plant types with a large gradient in canopy structure, i.e., sugar beet, winter wheat, and fruit trees. $f_{\mathrm{esc}}$ estimates of the three plant types were used to downscale HyPlant-derived SIF_760_ maps from canopy (${\mathrm{SIF}}_{760}^{\mathrm{canopy}}$) to leaf level (${\mathrm{SIF}}_{760}^{\mathrm{leaf}}$). Our findings provide important insights that facilitate the future development of methods to scale SIF_760_ from canopy to leaf level, yielding improved capabilities to interpret variations in plant photosynthesis in the spatial and temporal domains. This is especially important for satellites measuring SIF of entire ecosystems, such as the upcoming FLuorescence EXplorer (FLEX) mission of the European Space Agency (ESA) (Drusch et al., 2017, Mohammed et al., 2019).

## 2 Materials and methods

### 2.1 Study area

**Fig. 1:**
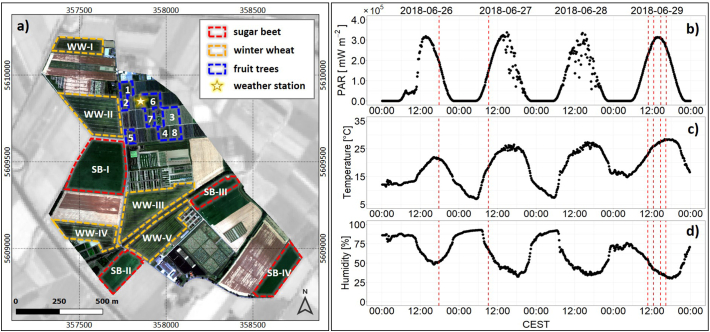
Airborne image of the agricultural research station Campus Klein-Altendorf and meteorological measurements recorded at the time of the overflights. (a) HyPlant DUAL top-of-canopy (TOC) true-colour composite (RGB 640/550/460 nm) of the campus acquired on June 29th 2018 with dashed lines highlighting the locations of the sugar beet (SB-I – IV, red) and winter wheat fields (WW-I – V, orange) as well as the fruit tree parcels (1–8, blue). Background: Sentinel-2 (Band 8) from June 27th 2018. (b) Photosynthetic active radiation (PAR), (c) air temperature and (d) relative humidity measurements recorded by the campus-internal weather station in the period from June 26th–29th 2018. The vertical red dashed lines indicate the time points of the six HyPlant overflights. (For interpretation of the references to colour in this figure legend, the reader is referred to the web version of this article.)

The data set presented in this study was acquired during the 2018 ESA FLEXSense campaign, a large field campaign conducted in preparation for the upcoming FLEX satellite mission. As one of the core test sites of this activity, the agricultural research station Campus Klein-Altendorf (CKA) was intensively investigated. CKA is one of the leading agricultural research facilities in Germany and is affiliated with the Agricultural Faculty of the University of Bonn. It is located in the western part of Germany (50°37′N, 6°59′E), 40 km south of Cologne between the towns of Meckenheim and Rheinbach, and covers an area of 181 ha for field trials. Beside the cultivation of typical regional crops, such as barley, wheat, sugar beet, and maize, the northeastern part of the area is used for growing fruit. The orchard offers space for many different apple, pear, and cherry species. Since data were acquired at the end of June 2018, the focus was on sugar beet, winter wheat, and fruit trees. At that time, these crops had a closed canopy and were still photosynthetically active. We deliberately included these structurally contrasting crop types to facilitate the assessment of structural effects on leaf-emitted SIF as retrieved at canopy scale. Fig. 1a shows the test site, while the investigated fields are highlighted with dashed lines. Parcels of the fruit orchard, which were covered with hail protection nets, were excluded from the analysis due to interference from the netting.

### 2.2 Investigated crops

A total of four sugar beet and five winter wheat fields were investigated and standard plant parameters were collected on the ground, e.g., growth stage, fractional cover, and plant height. The sugar beet fields had sizes varying from 2.63 to 8.83 ha, with sowing dates in early April 2018. During the HyPlant data acquisition, sugar beet was in growth stage 40 according to the scale of the Biologische Bundesanstalt, Bundessortenamt und CHemische Industrie (BBCH). During this stage, the plants covered more than 90% of the ground and had an average height of 50 cm. In general, sugar beet is characterized by a changing leaf orientation throughout the day, from predominantly erectophile in the morning to predominantly planophile in the afternoon (Danson and Aldakheel, 2000). This effect is caused by rising temperatures and irradiance over the course of the day and is therefore mainly pronounced during summer.

Winter wheat, however, has a spherical and constant leaf angle distribution throughout the day (Zou et al., 2014). The sowing of the investigated winter wheat fields took place between the end of October and the beginning of November 2017. Due to the different sowing dates, the growing stage of the plants in the different fields varied from BBCH stages 75 to 79, the principal stages of fruit development. The winter wheat fields varied in size between 2.99 and 6.11 ha and the plants had an average height of 87 cm during airborne data acquisition.

The investigated fruit tree parcels in the northeast of the study site were characterized by a more vertically pronounced canopy with complex crown structures of the single trees. This had a considerable influence on the SIF signal measured at canopy scale. Five of the eight investigated parcels were comprised of apple trees and three of pear trees. The tree ages of the different parcels varied between six month and four years, which led to substantial differences in the green biomass, LAI, and fractional cover of the investigated parcels. Further information about the different sugar beet and winter fields, and the fruit tree parcels, can be found in Fig. S1 in the supplemental section.

### 2.3 Meteorological data

Figs. 1b–d show the diurnal course of photosynthetic active radiation (PAR), air temperature, and relative humidity measured at the campus-internal weather station between June 26th and 29th, 2018. Whereas air temperature and relative humidity are only shown to illustrate the comparable conditions during the different days of HyPlant airborne data acquisition, PAR measurements were later used to normalize retrieved SIF.

PAR was measured with a DK-PHAR2 quantum sensor (deka Sensor + Technologie Entwicklungs- und Vertriebsgesellschaft mbH, Germany). The sensor records the photosynthetic photon flux density (PPFD) in micromoles per square meter per second

(μmol m^−2^ s^−1^). In remote sensing, however, it is more common to express light in energy instead of quantum units. For this reason, the PAR measurements were converted to watt per square meter (W m^−2^) using the approximate conversion factor 0.219 (1 μmol m^−2^ s^−1^ ≈ 0.219 W m^−2^) for PAR in the range of 400–700 nm (Reis and Ribeiro, 2020; Thimijan and Heins, 1983). Finally, PAR was further converted to milliwatts per square meter (mW m^−2^) to ensure it is comparable to the unit of SIF retrieved from HyPlant data.

### 2.4 Airborne data

Airborne data were acquired with the HyPlant imaging spectrometer, which was developed by Forschungszentrum Jülich in cooperation with Specim Ltd. (Finland) in 2012. The HyPlant sensor system consists of three pushbroom line scanners. Two scanners share the same optic and together form the DUAL module, which covers the visible/near-infrared (VNIR) and shortwave infrared (SWIR) range from 380 to 2500 nm with a full width at half maximum (FWHM) of 3.65 nm (VNIR) and 10.55 nm (SWIR), respectively. The second module, named FLUO, records image data in the NIR spectral range between 670 and 780 nm with a finer spectral resolution (FWHM = 0.28 nm) and high signal-to-noise ratio (SNR). The spectral characteristics of FLUO image data enable the retrieval of SIF in the O_2_-A and O_2_-B absorption features located at 760 and 687 nm, respectively. A detailed description of the HyPlant sensor system can be found in Siegmann et al. (2019) and Rascher et al. (2015).

In 2018, HyPlant was installed aboard a Cessna 208B Grand Caravan from the Global Change Research Institute (CzechGlobe). For three days, between June 26th and 29th, CKA was recorded six times at a flight altitude of 680 m above ground level, leading to a ground sampling distance (GSD) of 1 m. During each acquisition, four flight lines were alternately recorded towards either northwest or southeast directions to cover the entire area of CKA. The first two overflights took place on June 26th in the afternoon (17:15, Central European Summer Time (CEST)) and June 27th in the morning (10:10, CEST). Cloudy conditions on June 28th meant that data acquisition was continued on June 29th when CKA was recorded once in the morning (11:15, CEST), twice at midday (12:30 and 14:40, CEST) and once in the afternoon (15:50, CEST).

Using the six overflights, the study site was measured three times before and three times after local solar noon (13:30, CEST). HyPlant data acquisition (red dashed lines in Figs. 1b–d) always occurred under clear sky conditions, as illustrated by the diurnal patterns of the PAR curves for June 26th, 27th, and 29th in Fig. 1b.

Different processing steps were applied to the raw data acquired by the two HyPlant sensor modules. As a first step, HyPlant DUAL and FLUO data were radiometrically corrected using the CaliGeoPro software (Specim Ltd., Finland) to produce at-sensor radiance. The at-sensor radiance of the FLUO data formed the basis of the SIF retrieval, which is described in Section 2.5. The DUAL data were further atmospherically corrected with the ATCOR-4 software (ReSe Applications GmbH, Switzerland) to obtain TOC radiance and reflectance. Furthermore, different reflectance indices were calculated based on the HyPlant DUAL TOC reflectance images. Detailed information about the indices used in this study are provided in Sections 2.6 and 2.7.

During the acquisition of HyPlant DUAL or FLUO data, the exact location and orientation were measured with a Global Positioning System (GPS) and an Inertial Measurement Unit (IMU) to facilitate the geometric correction of the flight lines. Finally, single flight lines were mosaicked to create spatial maps covering the entire area of CKA. An extensive description of the HyPlant processing chain can be found in Siegmann et al. (2019).

### 2.5 HyPlant SIF retrieval

A new airborne-based implementation of the spectral fitting method (SFM), originally introduced by Cogliati et al. (2015) and adapted to enable a robust characterization of atmospheric interferences, was applied to retrieve SIF in-filling in both the O_2_-A and O_2_-B oxygen absorption bands. This method was applied to quantify SIF_760_ from HyPlant FLUO data in this study.

In brief, the SFM approach simulates at-sensor radiance spectra around the O_2_-A absorption band using a combined surface-atmospheric radiative transfer model. Atmospheric absorption and scattering effects are represented by transmittances, path radiance, and spherical albedo, and are simulated using the MODTRAN5 model (Berk et al., 2005). SIF and reflectance spectra are modeled using mathematical functions, i.e., polynomial and Gaussian-like functions. The final retrieval is based on finding the best fit between simulated and measured at-sensor radiances.

The new optimized approach to enable a robust characterization of atmospheric absorption and scattering effects, which uses the findings of Damm et al. (2014), exploits the entire image content, in particular the spectral information from non-vegetated pixels within the O_2_-A absorption band. The idea is to estimate an ‘effective’ surface-sensor distance, i.e., the geometric distance that results in a reproduction of the O_2_ absorption observed over non-vegetated surfaces. The approach allows for the indirect inclusion of the effect of atmospheric pressure within MODTRAN5 and, thus, a more accurate modeling of spectra in the range of the O_2_ absorption band. In practice, the method consists of: i) identification of non-fluorescent HyPlant pixels measured in nadir with a NDVI ≤ 0.1; ii) estimation of the ‘effective’ surface-sensor distance using a MODTRAN look-up table, resulting in zero SIF retrieval for non-vegetated pixels, and iii) decoupling of SIF and reflectance using the common SFM technique.

### 2.6 Estimation of fAPAR_green_ and fAPAR_chl_

The fraction of PAR absorbed by a vegetated surface is denoted as fAPAR and can be further divided into fAPAR_green_ (the fraction of PAR absorbed solely by the green leaf material of a canopy) and fAPAR_chl_ (the fraction of PAR absorbed by leaf chlorophyll). In the past, different approaches have been developed deriving fAPAR_green_ directly from optical remote sensing data. Different studies found that the NDVI is suited for use as a linear proxy for fAPAR_green_ estimates (Asrar et al., 1992, Liu et al., 2017). Since the NDVI is known to saturate in dense green vegetation (fAPAR_green_ > 0.7), the WDRVI developed by Gitelson (2004) was applied to overcome this problem. The WDRVI has shown a high sensitivity to the entire range of fAPAR_green_ (Viña and Gitelson, 2005). It can be calculated as:

(1) $$\text{WDRVI}=\frac{\alpha \;R_{795-810}-R_{665-680}}{\alpha \;R_{795-810}+R_{665-680}}$$

where $\;R_{795-810}$ and $R_{665-680}$ correspond to the average reflectance of the HyPlant DUAL spectral bands, covering the spectral ranges from 795 to 810 nm (NIR) and from 665 to 680 nm (red), respectively, and α is a weighting factor of 0.1 (Liu et al., 2019; Gitelson, 2004). The linear correlation between WDRVI and fAPAR_green_ was shown through model simulations using the Soil Canopy Observation, Photochemistry and Energy fluxes (SCOPE) model (van der Tol et al., 2009), considering a broad range of canopy and illumination representations (cf., Liu et al., 2019). Therefore, the linear equation determined by Liu et al. (2019) was used to estimate fAPAR_green_ based on WDRVI obtained from HyPlant DUAL data as:

(2) $${\text{fAPAR}}_{\text{green}}=0.516\;\text{WDRVI}+0.726\;$$

Subsequently, fAPAR_chl_ was computed using the following equation based on the findings of Du et al. (2017):

(3) $${\text{fAPAR}}_{\text{chl}}=k\times {\text{fAPAR}}_{\text{green}}\;$$

where k is a factor corresponding to the ratio of fAPAR_chl_ and fAPAR_green_. Du et al. (2017) estimated k from several SCOPE simulations and found that a k of 0.79 is a good representation of canopies with a leaf chlorophyll content (LCC) higher than 20 μg cm^−2^. A PROSAIL model (Jacquemoud et al., 2009) inversion of a HyPlant DUAL data set acquired on June 29th, 2018 (Fig. S2) and 25 field samples collected from sugar beet and winter wheat leaves within the study site on the same day (35–78 μg cm^−2^) provided leaf chlorophyll content values higher than 20 μg cm^−2^. k was therefore set to 0.79 in Eq. 3 to determine fAPAR_chl_. Finally, multiplying fAPAR_chl_ with PAR measured at the weather station (Section 2.2) enabled the calculation of the amount of photosynthetically active radiation absorbed by chlorophyll (APAR_chl_) for each crop on the pixel level.

### 2.7 Downscaling of SIF_760_ from canopy to leaf level

The FCVI, developed by Yang et al. (2020), is a surrogate of the product of fAPAR and $f_{\mathrm{esc}}$ of far-red SIF. This index is defined as the difference between NIR and broadband visible (VIS) reflectance acquired under a sun-canopy-observer geometry identical to that of the SIF measurements:

(4) $$\text{FCVI}=R_{\mathrm{NIR}}-R_{\bar{\mathrm{VIS}}}\approx \text{fAPAR}\times f_{\mathrm{esc}}$$

where $R_{\mathrm{NIR}}$ is the directional reflectance at the NIR plateau roughly stretching from 750 to 900 nm, close to the spectral band of interest for far-red SIF (760 nm). TOC reflectance at 770 nm was used because it is close to the band of interest but the effect of fluorescence on apparent TOC reflectance is negligible. $R_{\bar{\mathrm{VIS}}}$ corresponds to the broadband visible directional reflectance covering the spectral range of PAR from 400 to 700 nm. The FCVI was derived from HyPlant DUAL TOC reflectance data. Once Eq. 4 was rearranged, it was possible to determine $f_{\mathrm{esc}}$ for each pixel as the ratio of FCVI to ${\mathrm{fAPAR}}_{\mathrm{chl}}$:

(5) $$f_{\mathrm{esc}}\approx \frac{\text{FCVI}}{{\text{fAPAR}}_{\mathrm{chl}}}\;$$

Finally, the hemispherical ${\mathrm{SIF}}_{760}$ emission of all leaves within an observed pixel (${\mathrm{SIF}}_{760}^{\mathrm{leaf}}$) was calculated as the function of the directional ${\mathrm{SIF}}_{760}$ emission of the same pixel at canopy level (${\mathrm{SIF}}_{760}^{\mathrm{canopy}}$), π, and the escape fraction ($f_{\mathrm{esc}}$) obtained from Eq. 5 as:

(6) $${\mathrm{SIF}}_{760}^{\text{leaf}}=\frac{\pi \;{\mathrm{SIF}}_{760}^{\text{canopy}}}{f_{\mathrm{esc}}}\;$$

### 2.8 Normalization of $SIF_{760}^{leaf}$

Since ${\mathrm{SIF}}_{760}^{\mathrm{leaf}}$ is mainly driven by PAR throughout the day (Amoros-Lopez et al., 2008), two normalization schemes were applied to exclude the natural variations of incoming light on the leaf SIF emission signal. First, ${\mathrm{SIF}}_{760}^{\mathrm{leaf}}$ was normalized by PAR. This apparent SIF emission efficiency represents the total SIF emission at leaf level normalized by the total incoming PAR and is referred to as $\varepsilon_{\mathrm{SIF}(\mathrm{PAR})}$ in the further course of this study.

(7) $$\varepsilon_{\mathrm{SIF}\left(\mathrm{PAR}\right)}=\frac{{\mathrm{SIF}}_{760}^{\text{leaf}}}{\mathrm{PAR}}\;$$

Second, ${\mathrm{SIF}}_{760}^{\mathrm{leaf}}$ was normalized by APAR_chl_ (product of fAPAR_chl_ and PAR). This ratio was called $\varepsilon_{\mathrm{SIF}\left(\mathrm{APARchl}\right)}$ and can be calculated as:

(8) $$\varepsilon_{\mathrm{SIF}\left(\text{APARchl}\right)}=\frac{{\mathrm{SIF}}_{760}^{\text{leaf}}}{\text{fAPARchl}\times \mathrm{PAR}}$$

The advantage of this normalization procedure is that it can also be calculated without knowledge of fAPAR_chl_ and ${\mathrm{SIF}}_{760}^{\mathrm{leaf}}$. This is achieved by substituting $f_{\mathrm{esc}}$ in Eq. 6 with the ratio of FCVI to fAPAR_chl_ from Eq. 5:

(9) $${\mathrm{SIF}}_{760}^{\text{leaf}}\approx \frac{\pi \;{\mathrm{SIF}}_{760}^{\text{canopy}}\times {\text{fAPAR}}_{\mathrm{chl}}}{\text{FCVI}\;}\;$$

Using the right side of Eq. 9 to represent ${\mathrm{SIF}}_{760}^{\mathrm{leaf}}$ in Eq. 8, ${\mathrm{fAPAR}}_{\mathrm{chl}}$ is eliminated and $\varepsilon_{\mathrm{SIF}\left(\mathrm{APARchl}\right)}$, which is called $\varepsilon_{\mathrm{SIF}\left(\mathrm{FCVI}\right)}\;$in the further course of this study, can be expressed as follows:

(10) $$\varepsilon_{\mathrm{SIF}\left(\text{APARchl}\right)}\approx \varepsilon_{\mathrm{SIF}\left(\text{FCVI}\right)}=\frac{\pi \;{\mathrm{SIF}}_{760}^{\text{canopy}}}{\text{FCVI}\times \mathrm{PAR}}$$

## 3 Results

### 3.1 *Diurnal course of*$SIF_{760}^{\mathrm{canopy}}$

**Fig. 2:**
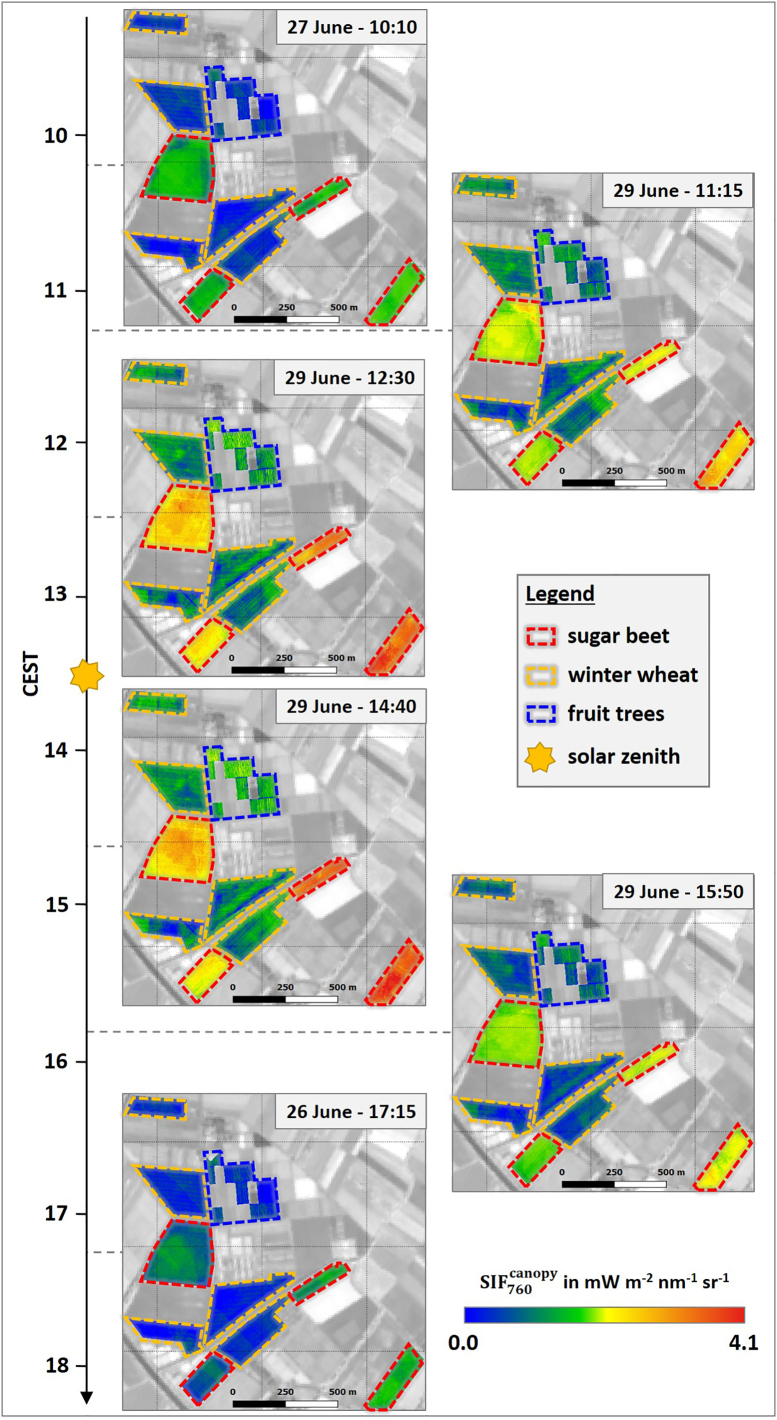
Spatial dynamics of canopy SIF_760_ (${\mathrm{SIF}}_{760}^{\mathrm{canopy}}$) of different crops in the course of the day. The dashed lines highlight the locations of the investigated sugar beet (red) and winter wheat fields (orange) as well as the investigated parcels of the fruit orchard (blue). Background: Sentinel-2 (Band 8) from June 27th 2018. (For interpretation of the references to colour in this figure legend, the reader is referred to the web version of this article.)

**Fig. 3:**
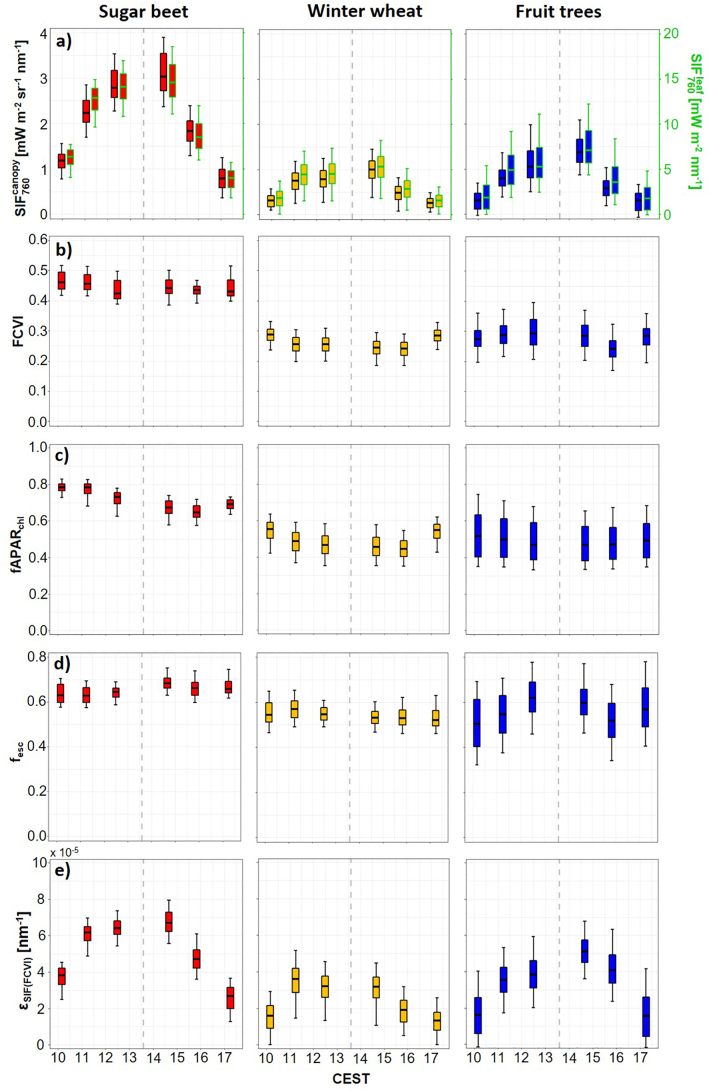
Box plot of the median, 0th, 25th, 75th and 100th percentiles showing the diurnal course of canopy (${\mathrm{SIF}}_{760}^{\mathrm{canopy}}$) and leaf SIF_760_ (${\mathrm{SIF}}_{760}^{\mathrm{leaf}}$) (a), fluorescence correction vegetation index (FCVI) (b), fraction of absorbed photosynthetically active radiation by leaf chlorophyll (fAPAR_chl_) (c), SIF_760_ escape fraction (f_esc_) (d) and SIF_760_ emission efficiency as a function of APAR_chl_ ($\varepsilon_{\mathrm{SIF}(\mathrm{FCVI})}$) (e) of sugar beet (red), winter wheat (orange) and fruit trees (blue). The vertical dashed grey lines indicate the time of solar culmination. (For interpretation of the references to colour in this figure legend, the reader is referred to the web version of this article.)

Fig. 2 illustrates the spatial dynamics of ${\mathrm{SIF}}_{760}^{\mathrm{canopy}}$ over the course of the day for the investigated crops. All three plant types exhibited the typical diurnal behavior following the intensity of PAR (Fig. 1b) with rising values from the morning until solar noon (13:30) and a decrease of SIF in the afternoon. This typical diurnal pattern is also visible in Fig. 3a, where ${\mathrm{SIF}}_{760}^{\mathrm{canopy}}$ of the different crops is displayed in the form of box plots for each HyPlant overflight. During the day, sugar beet showed the highest ${\mathrm{SIF}}_{760}^{\mathrm{canopy}}$ with values of approximately 1 mWm^−2^nm^−1^sr^−1^ in the morning and afternoon, and around 3 mWm^−2^nm^−1^sr^−1^ close to solar noon. In contrast, winter wheat fields and fruit trees had distinctly lower values varying in the range of 0.4–1 mWm^−2^nm^−1^sr^−1^. While the observed spatial ${\mathrm{SIF}}_{760}^{\mathrm{canopy}}$ variability in winter wheat was relatively small, it was much more pronounced in sugar beet and fruit trees (Fig. 3a). This is further confirmed by the ${\mathrm{SIF}}_{760}^{\mathrm{canopy}}$ maps in Fig. 2, where the inter-field variability of winter wheat was much lower in comparison to that of sugar beet. Both sugar beet fields in the eastern part of the study area had distinctly higher ${\mathrm{SIF}}_{760}^{\mathrm{canopy}}$ values compared to the two fields in the western part, particularly around solar noon. In contrast, the intra-field variability of wheat fields appeared to be slightly higher than the variability of sugar beet fields, partly due to the visibility of the tractor trails within the wheat fields where no plants grow. The orchard in the northeastern part of the study site is a special case since different species and trees of different ages were grown there. However, two parcels in the northern part of the area (marked with the blue dashed lines in Fig. 2) showed higher values in the midday overpasses in comparison to the rest of the observed orchard. These two parcels comprised apple trees and pear trees, which had the highest age (four years) of all investigated trees (Fig. S1).

### 3.2 *Diurnal course of FCVI and*$f_{esc}$

The diurnal trends in FCVI of sugar beet and winter wheat were similar. For both crops, a slight decline of values was observed in the first two overflights in the morning (10:10, 11:15). At midday (overflights at 12:30 and 14:40), the FCVI remained stable before it further decreased in the early afternoon (overflight at 15:50), followed by an increase in the late afternoon (overflight at 17:15) (Fig. 3b).

While the FCVI of winter wheat was lower on average than that of sugar beet, the investigated fruit trees provided a similar value range to winter wheat but with a higher degree of variance. Additionally, the diurnal pattern of the fruit trees was different from the other crops, characterized by an increase of FCVI before solar noon followed by a decrease during the two afternoon overflights (14:40 and 15:50).Subsequently, in comparison to sugar beet and winter wheat, the FCVI increased slightly in the last data set (17:15).

**Fig. 4:**
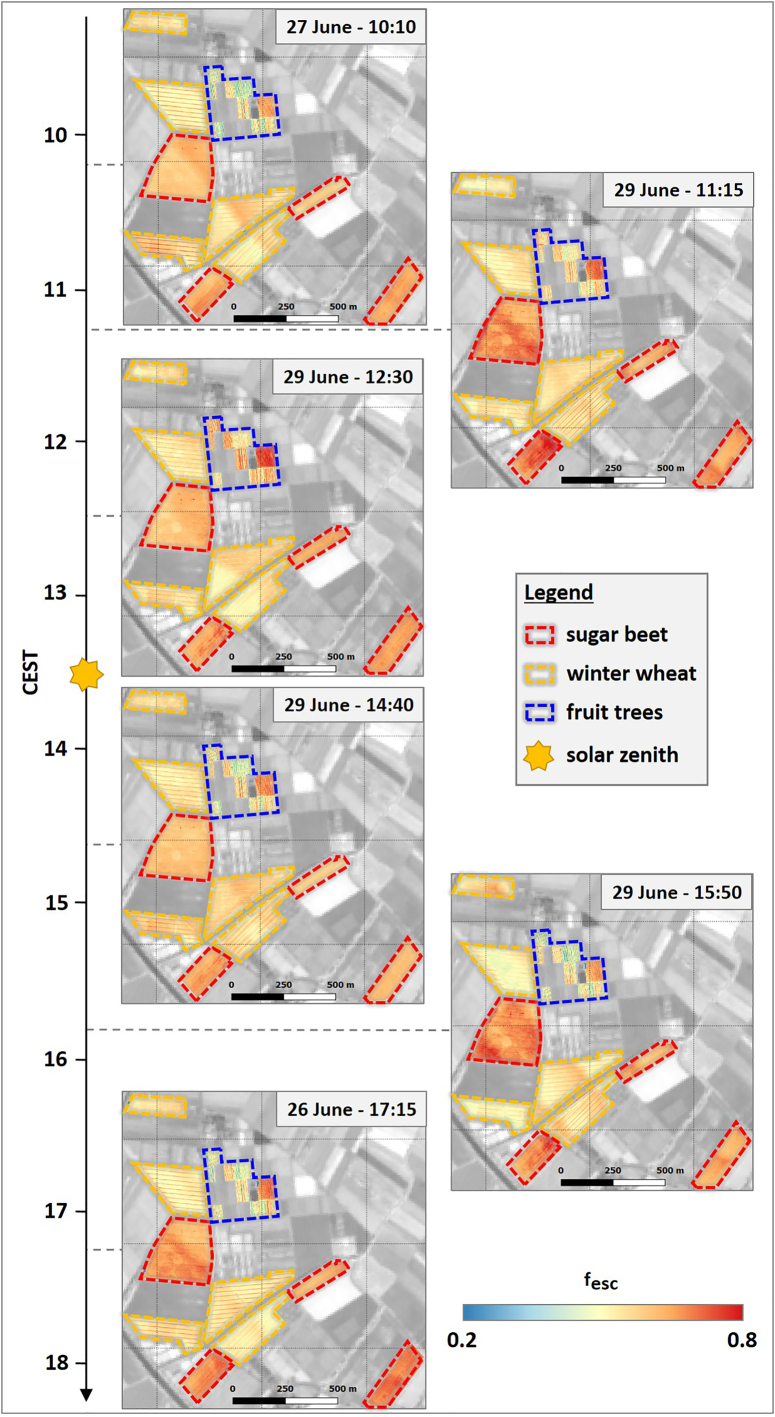
Spatial dynamic of the SIF_760_ escape fraction (f_esc_) of different crops in the course of the day. The dashed lines highlight the locations of the investigated sugar beet (red) and winter wheat fields (orange) as well as the investigated parcels of the fruit orchard (blue). Background: Sentinel-2 (Band 8) from June 27th 2018. (For interpretation of the references to colour in this figure legend, the reader is referred to the web version of this article.)

Fig. 3d shows the diurnal course of $f_{\mathrm{esc}}$ of sugar beet, winter wheat, and fruit trees. It can be clearly seen that $f_{\mathrm{esc}}$ has a low variability over the course of the day. While $f_{\mathrm{esc}}$ of winter wheat was at the same level throughout the entire day (approximately 0.55), sugar beet showed a diurnal trend with increasing $f_{\mathrm{esc}}\;$from the first (10:10) to the fourth overflight (14:40), followed by a rather constant $f_{\mathrm{esc}}\;$at 15:50 and 17:15. In general, $f_{\mathrm{esc}}$ of sugar beet was higher (ranging from 0.6 to 0.7) than $f_{\mathrm{esc}}\;$of winter wheat. This is underlined by the $f_{\mathrm{esc}}$ maps presented in Fig. 4, in which the sugar beet fields have clearly higher values in comparison to the winter wheat fields. The intra-field variability in the $f_{\mathrm{esc}}$ maps was relatively low, indicating homogenous field conditions and reflecting the low variance in $f_{\mathrm{esc}}$, as illustrated by the box plots in Fig. 3d. The harsh transitions from lower to higher values, visible in some sugar beet and winter wheat fields in Fig. 4, e.g., in the overflight at 10:10, corresponds to the border areas of adjacent HyPlant flight lines.

In contrast to sugar beet and winter wheat, the observed fruit tree parcels showed distinct spatial differences in $f_{\mathrm{esc}}$ throughout the day (Fig. 4). Of particular note is one parcel with a very high $f_{\mathrm{esc}}$ in the eastern part of the orchard in Fig. 4. This parcel is covered by young trees and thus had a low fractional cover (Fig. S1). The heterogeneity in $f_{\mathrm{esc}}$ of the different fruit tree parcels therefore explained the higher variance compared to sugar beet and winter wheat, as depicted in the box plots in Fig. 3d.

Another interesting fact is that the diurnal trends of f_esc_ of sugar beet and fruit trees (Fig. 3d) are very similar to the diurnal trends of the FCVI of both crops (Fig. 3b). For winter wheat, the same agreement is not visible, but instead the diurnal trend of FCVI and fAPAR_chl_ (Figs. 3b and c) is very similar.

### 3.3 Diurnal course of $SIF_{760}^{leaf}$

**Fig. 5:**
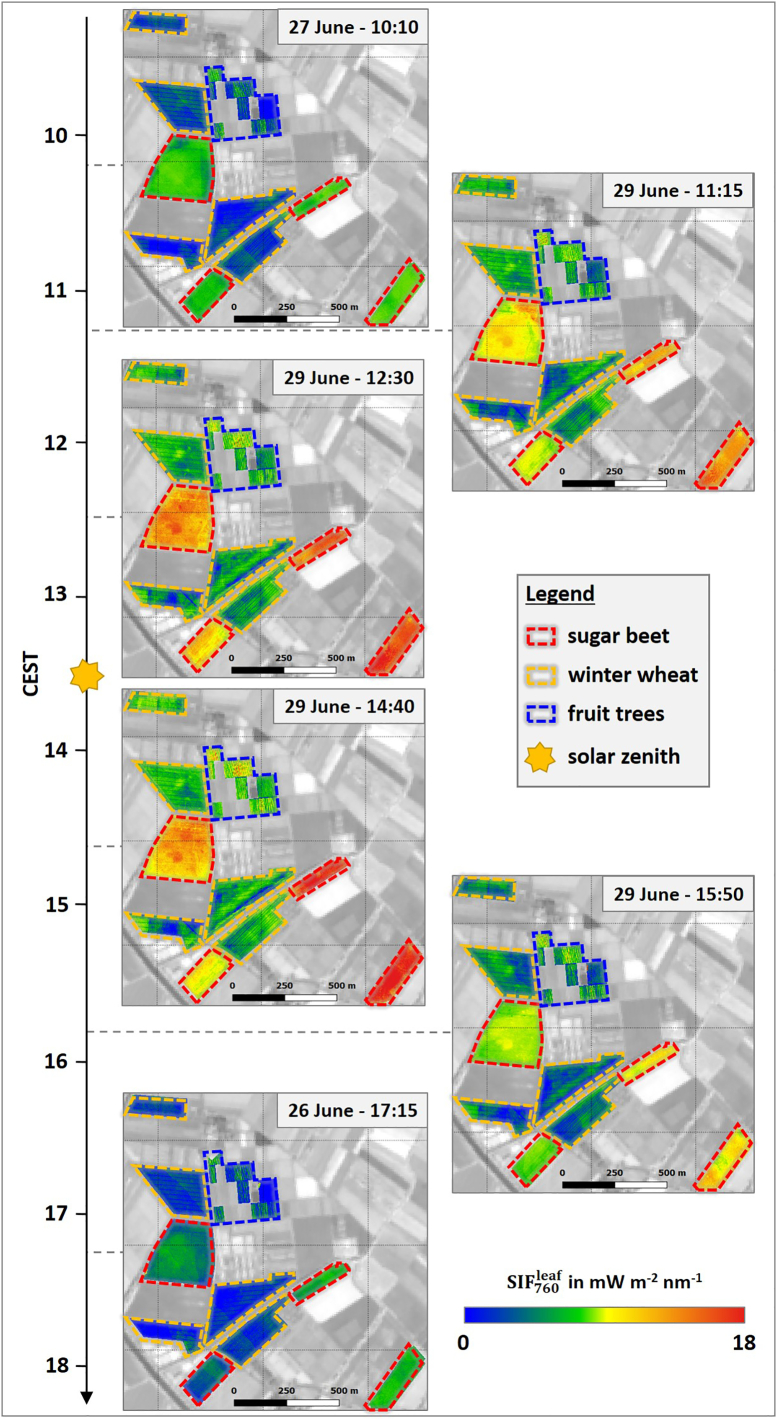
Spatial dynamics of leaf SIF_760_ (${\mathrm{SIF}}_{760}^{\mathrm{leaf}}$) of different crops in the course of the day. The dashed lines highlight the locations of the investigated sugar beet (red) and winter wheat fields (orange) as well as the investigated parcels of the fruit orchard (blue). Background: Sentinel-2 (Band 8) from June 27th 2018. (For interpretation of the references to colour in this figure legend, the reader is referred to the web version of this article.)

Diurnal trends of ${\mathrm{SIF}}_{760}^{\mathrm{leaf}}$ are presented in Fig. 3a in addition to ${\mathrm{SIF}}_{760}^{\mathrm{canopy}}$. The observed patterns of the trends at leaf and canopy level are very similar, which is related to only slight fluctuations in $f_{\mathrm{esc}}$ of the observed crops throughout the day. Since ${\mathrm{SIF}}_{760}^{\mathrm{leaf}}$ of the different plant types is also driven by the amount of incoming PAR, its diurnal trend was also characterized by an increase in the morning until solar noon and a subsequent decrease in the afternoon. Sugar beet again had the highest values varying from lower than 7.5 mWm^−2^ nm^−1^ in the morning and afternoon up to a maximum value of 18 mWm^−2^ nm^−1^ obtained from the overflight at 14:40. In contrast, similar to the canopy measurements, ${\mathrm{SIF}}_{760}^{\mathrm{leaf}}$ values determined from winter wheat and fruits trees were distinctly lower. On average, fruit trees showed slightly higher values (0–12 mWm^−2^ nm^−1^) and a more pronounced variance compared to winter wheat (0–8 mWm^−2^ nm^−1^). The similarity in the diurnal trends of SIF_760_ determined at canopy and leaf levels is additionally reflected in the high similarity of the associated maps shown in Fig. 2, Fig. 5.

**Fig. 6:**
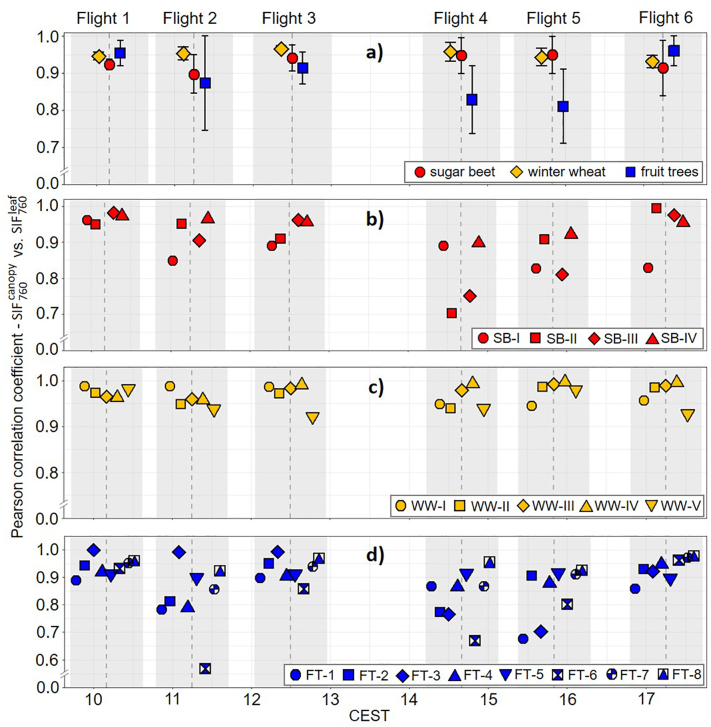
Correlation coefficients calculated for corresponding pixels of the canopy (${\mathrm{SIF}}_{760}^{\mathrm{canopy}}$) and leaf SIF_760_ (${\mathrm{SIF}}_{760}^{\mathrm{leaf}}$) maps derived from the six HyPlant overflights. Averaged correlation coefficients and standard deviations calculated for the three observed crops (sugar beet, winter wheat and fruit trees) (a), correlation coefficients for the individual sugar beet fields (SB-I – SB-IV) (b), correlation coefficients for the individual winter wheat fields (WW-I – WW-V) (c) and correlation coefficients for the individual fruit tree parcels (FT-1 – FT-8) (d). The vertical grey dashed lines indicate the time points of the six HyPlant overflights. The symbols used for the different crop fields and parcels were plotted with slight time offsets for a better overview. The light grey bars indicate to which overflights the different symbols belong.

A correlation analysis of the ${\mathrm{SIF}}_{760}^{\mathrm{canopy}}$ and ${\mathrm{SIF}}_{760}^{\mathrm{leaf}}$ maps is presented in Fig. 6. While Fig. 6a and Table S1 show the correlation coefficients for each crop separately, Figs. 6b–d and Tables S2–S4 provide information about the individual sugar beet and winter wheat fields as well as the fruit tree parcels. For winter wheat, the correlation coefficients at crop and field levels were very high (Figs. 6 a and c). This once again clearly illustrates the high level of agreement between ${\mathrm{SIF}}_{760}^{\mathrm{canopy}}$ and ${\mathrm{SIF}}_{760}^{\mathrm{leaf}}$ of this crop. In contrast to winter wheat, the correlation coefficients determined for sugar beet and fruit trees showed greater variability. This was particularly clear in the afternoon overflights in which some sugar beet fields and fruit tree parcels provided distinctly lower correlation coefficients (Figs. 6b and d).

**Fig. 7:**
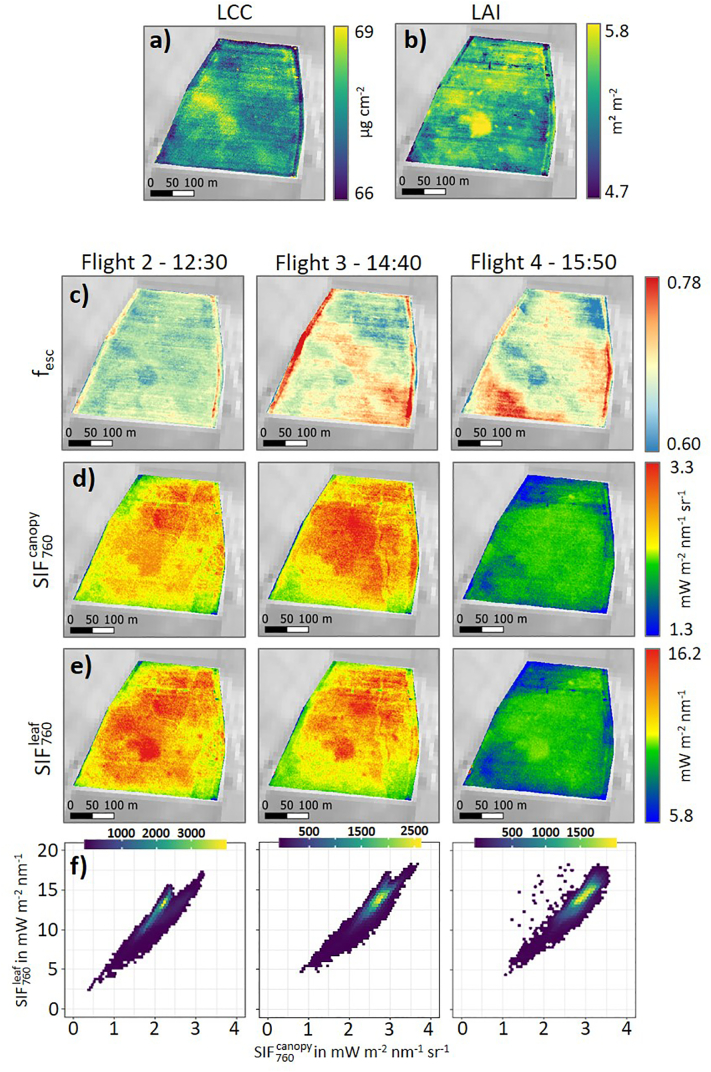
Spatial and temporal dynamics of sugar beet field SB-I. Leaf chlorophyll content (LCC) (a) and leaf area index (LAI) map (b) derived from HyPlant DUAL data recorded at 12:30. SIF_760_ escape fraction (f_esc_) derived from HyPlant DUAL data recorded at 12:30, 14:40 and 15:50 (c). Canopy (${\mathrm{SIF}}_{760}^{\mathrm{canopy}}$) (d) and leaf SIF_760_ (${\mathrm{SIF}}_{760}^{\mathrm{leaf}}$) maps (e) derived from HyPlant FLUO data recorded at 12:30, 14:40 and 15:50. Scatterplots of ${\mathrm{SIF}}_{760}^{\mathrm{canopy}}$ and ${\mathrm{SIF}}_{760}^{\mathrm{leaf}}$ for the three time points (f). Background of the maps: Sentinel-2 (Band 8) from June 27th 2018.

One of the sugar beet fields characterized by varying correlation coefficients throughout the day (SB-1) is shown in Fig. 7. Although the spatial patterns of the ${\mathrm{SIF}}_{760}^{\mathrm{canopy}}$ (Fig. 7d) and ${\mathrm{SIF}}_{760}^{\mathrm{leaf}}$ maps (Fig. 7e) of the three selected overflights (12:30, 14:40, and 15:50), show a high level of agreement, a roundish pattern in the southern central part of the field is only visible in the ${\mathrm{SIF}}_{760}^{\mathrm{leaf}}$ maps. The same roundish pattern is also detectable in the f_esc_ maps in form of lower values calculated from the three overflights (Fig. 7c) and corresponds to an area characterized by a high LCC and LAI (Figs. 7a and b).

### 3.4 Diurnal course of normalized $SIF_{760}^{leaf}$

Since ${\mathrm{SIF}}_{760}^{\mathrm{leaf}}$ determined for the three plant types in this study was mainly driven by PAR throughout the day (Fig. 3a), $\varepsilon_{\mathrm{SIF}\left(\mathrm{PAR}\right)}$ was calculated to normalize ${\mathrm{SIF}}_{760}^{\mathrm{leaf}}$ and thus exclude the natural variations of incoming light. Additionally, $\varepsilon_{\mathrm{SIF}\left(\mathrm{FCVI}\right)}$was calculated, which made a second normalization of ${\mathrm{SIF}}_{760}^{\mathrm{leaf}}$ possible by considering only the fraction of PAR absorbed by the chlorophyll within the leaves. Fig. S3 depicts the development of ${\mathrm{APAR}}_{\mathrm{chl}}$ in comparison to PAR for the three crops throughout the day. In general, ${\mathrm{APAR}}_{\mathrm{chl}}$ of all crops showed the same diurnal behavior as PAR. Sugar beet, however, was characterized by lower ${\mathrm{APAR}}_{\mathrm{chl}}$ values in the afternoon in comparison to the morning. This is further clarified in Fig. 3c, which depicts ${\mathrm{fAPAR}}_{\mathrm{chl}}$ of the observed plant types. In addition, it becomes clear that ${\mathrm{fAPAR}}_{\mathrm{chl}}$ derived for the winter wheat fields and fruit trees was distinctly lower than for the sugar beet fields.

**Fig. 8:**
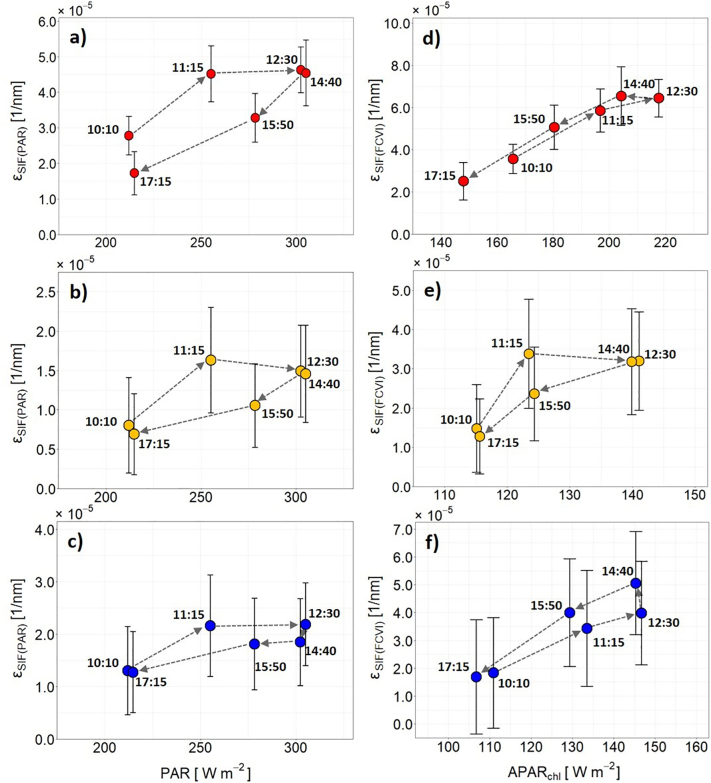
Plots of mean and standard deviation showing the diurnal dynamic of SIF_760_ emission efficiency as a function of PAR ($\varepsilon_{\mathrm{SIF}\left(\mathrm{PAR}\right)}$) (a-c) and as a function of ${\mathrm{APAR}}_{\mathrm{chl}}$ ($\varepsilon_{\mathrm{SIF}\left(\mathrm{FCVI}\right)}$) (d-f) for sugar beet (red), winter wheat (orange) and fruit trees (blue). (For interpretation of the references to colour in this figure legend, the reader is referred to the web version of this article.)

The diurnal behavior of $\varepsilon_{\mathrm{SIF}\left(\mathrm{PAR}\right)}$ is illustrated in Figs. 8a–c. For all three plant types, $\varepsilon_{\mathrm{SIF}\left(\mathrm{PAR}\right)}$ plotted as a function of PAR showed a clear hysteresis characterized by increasing values in the morning, from the first (10:10) to the second overflight (11:15) and remained constant between the second (11:15) and the third overflight (12:30). In the afternoon, the three plant types were characterized by declining values and again showed a positive correlation with PAR, but on a lower level compared to the morning increase. During the last overflight (17:15), $\varepsilon_{\mathrm{SIF}\left(\mathrm{PAR}\right)}$ returned to its initial values from the morning except for sugar beet, which provided $\varepsilon_{\mathrm{SIF}\left(\mathrm{PAR}\right)}$ on a lower level. Ultimately, all three plant types showed a comparable hysteresis in their diurnal relationships between PAR and $\varepsilon_{\mathrm{SIF}\left(\mathrm{PAR}\right)}$.

**Fig. 9:**
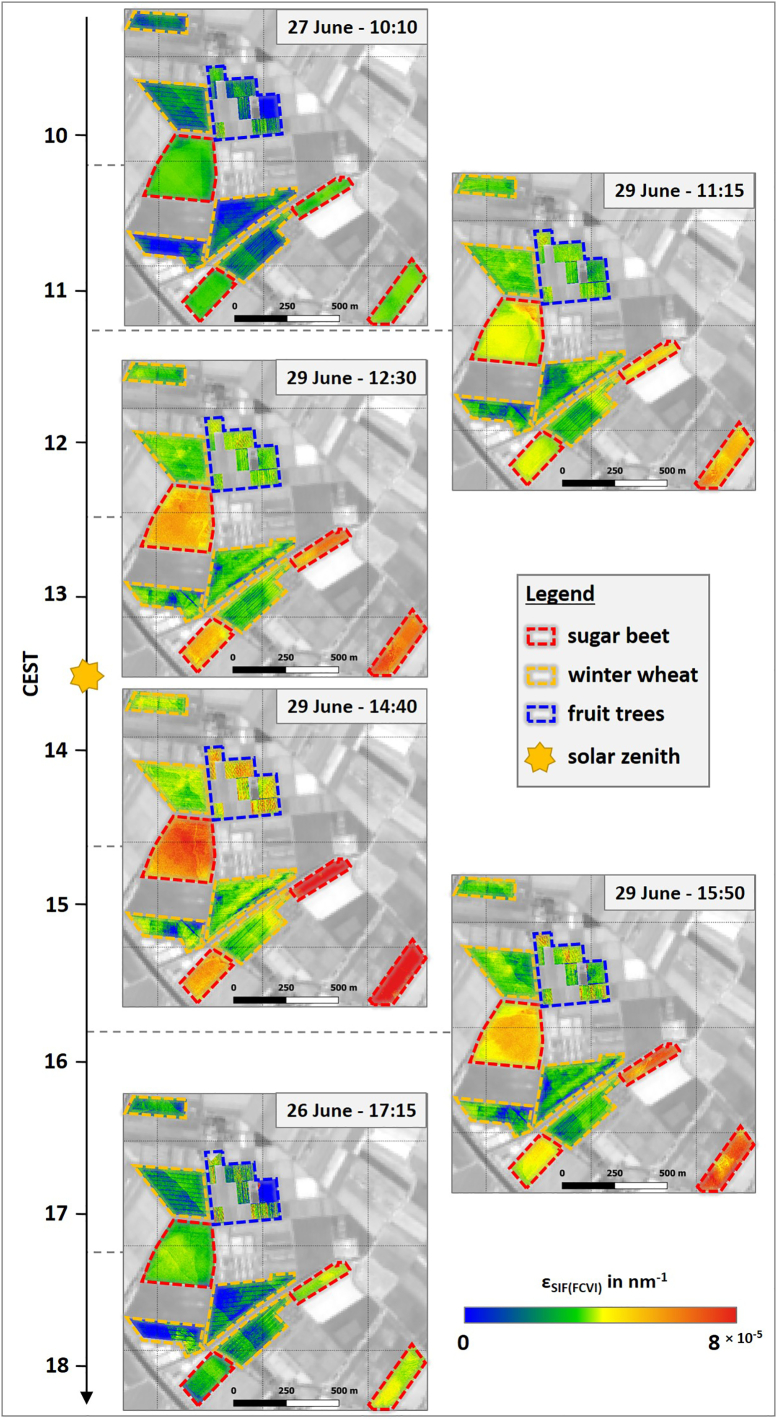
Spatial dynamics of the SIF_760_ emission efficiency as a function of APAR_chl_ ($\varepsilon_{\mathrm{SIF}\left(\mathrm{FCVI}\right)}$) of different crops in the course of the day. The dashed lines highlight the locations of the investigated sugar beet (red) and winter wheat fields (orange) as well as the investigated parcels of the fruit orchard (blue). Background: Sentinel-2 (Band 8) from June 27th 2018. (For interpretation of the references to colour in this figure legend, the reader is referred to the web version of this article.)

Plotting $\varepsilon_{\mathrm{SIF}\left(\mathrm{FCVI}\right)}$ as a function of APAR_chl_ led to different hysteresis (Figs. 8d, e, and f). Sugar beet and fruit trees had a positive correlation in the form of increasing values in the morning until 12:30 (third overflight). Thereafter, $\varepsilon_{\mathrm{SIF}\left(\mathrm{FCVI}\right)}$ of sugar beet remained stable until 14:40 (fourth overflight), while the fruit trees showed a slight increase in $\varepsilon_{\mathrm{SIF}\left(\mathrm{FCVI}\right)}$. In contrast, $\varepsilon_{\mathrm{SIF}\left(\mathrm{FCVI}\right)}$ of winter wheat only increased until 11:15 (second overflight) and remained constant until 14:40 (fourth overflight). During the afternoon overflights from 14:40 to 17:15, all plant types were characterized by a decline in $\varepsilon_{\mathrm{SIF}\left(\mathrm{FCVI}\right)}$, which in combination with decreasing ${\mathrm{APAR}}_{\mathrm{chl}}$ again resulted in a positive correlation between both parameters. Looking at the diurnal course as a whole, the trajectories of sugar beet and the fruit trees were similar and there was no, or only slight, hysteresis. In contrast, winter wheat still showed a clear hysteresis in the $\varepsilon_{\mathrm{SIF}\left(\mathrm{FCVI}\right)}$ APAR_chl_ diurnal relationship (Fig. 8e). The same diurnal trend is visible in Fig. 3e, in which $\varepsilon_{\mathrm{SIF}\left(\mathrm{FCVI}\right)}$ is plotted as a function of time. The diurnal course of sugar beet and fruit trees was characterized by increasing values in the morning and decreasing values in the afternoon. Both trends were similar to those observed for ${\mathrm{SIF}}_{760}^{\mathrm{canopy}}$ and ${\mathrm{SIF}}_{760}^{\mathrm{leaf}}$ (Fig. 3a). In contrast, winter wheat only showed a short steep increase from 10:10 (first overflight) to 11:15 (second overflight) before $\varepsilon_{\mathrm{SIF}\left(\mathrm{FCVI}\right)}$ started to decrease until 17:15 (sixth overflight). This diurnal trend in $\varepsilon_{\mathrm{SIF}\left(\mathrm{FCVI}\right)}$ is clearly different from the diurnal behavior of ${\mathrm{SIF}}_{760}^{\mathrm{canopy}}$ and ${\mathrm{SIF}}_{760}^{\mathrm{leaf}}$ (Fig. 3a). In addition, Fig. 9 shows the $\varepsilon_{\mathrm{SIF}\left(\mathrm{FCVI}\right)}$ maps that were derived from the six overflights. Compared to the ${\mathrm{SIF}}_{760}^{\mathrm{leaf}}$ maps presented in Fig. 5, the $\varepsilon_{\mathrm{SIF}\left(\mathrm{FCVI}\right)}$ maps of the three crops provided similar spatial patterns and comparable inter-field and intra-field variabilities. The roundish pattern in sugar beet field SB-I, for example, is again clearly visible in the two overflights before and after solar noon.

## 4 Discussion

### 4.1 Spatio-temporal dynamics of structural impact on SIF

In this study, a unique diurnal data set was used to assess structural interferences on the relation between leaf- and canopy-level SIF_760_ including underlying spatial and temporal dynamics. These novel insights can only be partly evaluated against existing results that solely address either the spatial or temporal dimension of this problem.

In general, observed diurnal trends of ${\mathrm{SIF}}_{760}^{\mathrm{canopy}}$ and ${\mathrm{SIF}}_{760}^{\mathrm{leaf}}$ for all crops followed the temporal dynamic of PAR. This is in accordance with previous studies presenting diurnal courses of SIF_760_ of different plants at canopy (Campbell et al., 2019; Liu et al., 2017) and leaf level (Süß et al., 2016; Amoros-Lopez et al., 2008). In addition, several studies reported data ranges of ${\mathrm{SIF}}_{760}^{\mathrm{canopy}}$ (Liu et al., 2019; Pinto et al., 2017; Rossini et al., 2015; Wieneke et al., 2016) comparable to those shown here. A validation of derived ${\mathrm{SIF}}_{760}^{\mathrm{leaf}}$ maps is more complex due to a lack of corresponding results. Several studies have already presented f_esc_ and corresponding ${\mathrm{SIF}}_{760}^{\mathrm{leaf}}$ maps derived from satellite data and thus shown the spatial variability of both parameters on a global scale (Qiu et al., 2019; Zhang et al., 2019). However, so far, only one study (Liu et al., 2019) has presented an airborne ${\mathrm{SIF}}_{760}^{\mathrm{leaf}}$ map that was also derived from HyPlant FLUO data. In contrast to the results of this study, Liu et al. (2019) used concepts developed by Yang and van der Tol (2018) in combination with random forest regression to derive $f_{\mathrm{esc}}$ from TOC reflectance data. A comparison of the published ${\mathrm{SIF}}_{760}^{\mathrm{leaf}}$ maps with our results derived from the overflight at 14:40 was considered appropriate, since both maps cover comparable agricultural regions in Germany and were acquired at almost the same time of day and the same day of the year. The observed data ranges were the same (0–18 mWm^−2^nm^−1^sr^−1^) and sugar beet had distinctly higher values compared to winter wheat in both results. These commonalities point to the reliability of the presented downscaling approach and demonstrate the plausibility of the derived ${\mathrm{SIF}}_{760}^{\mathrm{leaf}}$ maps.

The use of FCVI seems to represent a reliable strategy to estimate $f_{\mathrm{esc}}$ and downscale SIF_760_ from canopy to leaf level. Despite the anticipated low spatial variability of $f_{\mathrm{esc}}$ for sugar beet and winter wheat due to homogeneous field conditions, it must be noted that the diurnal/temporal variability of $f_{\mathrm{esc}}$ for sugar beet was lower than expected (Fig. 3d). This is surprising, since sugar beet is characterized by distinct changes in leaf orientation, from predominantly erectophile in the morning to predominantly planophile in the afternoon (Danson and Aldakheel, 2000). This diurnal leaf movement was only apparent by slightly higher f_esc_ values in the afternoon. During this time of the day, when temperatures reach their maximum, sugar beet plants tend to lay down their leaf rosette to prevent excessive water loss. For winter wheat, which is characterized by an almost spherical and constant leaf angle distribution across the day (Zou et al., 2014), the temporal dynamics matched our expectation with temporarily stable $f_{\mathrm{esc}}$ values (Fig. 3d). Fruit trees showed a more pronounced spatial and temporal variation of $f_{\mathrm{esc}}$. This was expected because the investigated tree stands covered different tree species (apple and pear) with varying ages (Fig. S1). The pronounced diurnal variability compared to sugar beet and winter wheat was likely caused by the clear row plantation, complex crown geometry of the trees, and changing illumination conditions: around solar noon, SIF_760_ measured by HyPlant mainly corresponded to fluorescence exited and emitted from leaves at the top of the canopy (Porcar-Castell et al., 2014), while lower layers were more shaded and contributed less to the total SIF radiance. Accordingly, scattering effects were less pronounced around noon, resulting in higher f_esc_ values (Fig. 3d). In contrast, higher solar zenith angles in the morning and evening hours resulted in more even illumination among higher and lower canopy layers. This caused stronger scattering in the entire canopy and thus led to higher diurnal dynamics in $f_{\mathrm{esc}}$.

Based on the diurnal analysis of winter wheat and sugar beet in the observed phenological stages, calculating $f_{\mathrm{esc}}$ only once a day for winter wheat and twice or three times a day for sugar beet would have been sufficient to scale far-red SIF from canopy to leaf level. In addition, the low spatial variability of winter wheat in $f_{\mathrm{esc}}$ could be the reason for the high similarity of corresponding ${\mathrm{SIF}}_{760}^{\mathrm{leaf}}$ and ${\mathrm{SIF}}_{760}^{\mathrm{canopy}}$maps (Fig. 6c). For sugar beet, the spatial variability was also very low in the morning but much more pronounced in the afternoon, which could again be related to changes in leaf orientation throughout the day (Fig. 6b). The different parcels of the orchard also exhibited clear spatial differences in corresponding far-red SIF maps derived for the two different scales (Fig. 6d). This was due to the change in $f_{\mathrm{esc}}$ throughout the day as well as the different age and canopy structure of the trees. For this reason, determining $f_{\mathrm{esc}}$ of the fruit trees from each overflight was critical for the downscaling of far-red SIF to leaf level.

The ${\mathrm{SIF}}_{760}^{\mathrm{leaf}}$ maps in Fig. 5 showed some harsh transition between adjacent flight lines that were possibly caused by bidirectional effects (especially in the morning and afternoon overflights). Although the FCVI accounts for those effects, the anisotropic response of TOC reflectance that was used to determine fAPAR_chl_, and in turn, $f_{\mathrm{esc}}$ could be a possible explanation for the clearly visible differences within the fields, which were covered by two flight lines. In addition, as was recently reported by Biriukova et al. (2020), the anisotropic response is different for reflectance and SIF at canopy level, which could explain the more pronounced bidirectional effects in the $f_{\mathrm{esc}}$ (Fig. 4) in comparison to the ${\mathrm{SIF}}_{760}^{\mathrm{canopy}}$ maps (Fig. 2).

The spatio-temporal differences of f_esc_ derived for the three investigated crops clearly indicate the strong influence of canopy geometry on ${\mathrm{SIF}}_{760}^{\mathrm{canopy}}$ measurements. A crop-specific determination of f_esc_ is therefore critical to successfully downscaling SIF_760_ from canopy to leaf level. This is especially important when SIF is used to estimate GPP. While recent studies by Zhang et al., 2019, Zhang et al., 2020 based on site-specific and global SIF measurements have demonstrated an improved relationship between SIF_760_ and GPP for numerous plant types when ${\mathrm{SIF}}_{760}^{\mathrm{canopy}}$ was corrected for f_esc,_ Dechant et al. (2020) determined a decreased correlation between SIF_760_ and GPP for tower-based SIF measurements of different crops when f_esc_ was applied to downscale SIF_760_ to the leaf level. These inconsistent results illustrate that more research is needed to better understand the influence of the canopy structure on the relationship between SIF_760_ and GPP. This is also of particular importance to further improve GPP estimates from HyPlant ${\mathrm{SIF}}_{760}^{\mathrm{canopy}}$measurements, as presented in Tagliabue et al. (2019) and Wieneke et al. (2016).

### 4.2 Physiological interpretation of SIF

Correcting canopy structural effects and thus linking canopy- and leaf-level SIF_760_ is essential to tracking physiological canopy responses using HyPlant-based SIF retrievals. The normalization of ${\mathrm{SIF}}_{760}^{\mathrm{leaf}}$ for dynamics in illumination conditions, i.e. PAR and ${\mathrm{APAR}}_{\mathrm{chl}}$, to eventually obtain $\varepsilon_{\mathrm{SIF}}$ (often referred to as SIF yield) is considered important to estimate the photosynthetic light use efficiency (LUE) (Mohammed et al., 2019, Wieneke et al., 2018). Although recent studies have reported moderate to high correlations in the seasonal dynamics of f_esc_ and LUE (Dechant et al., 2020; Liu et al., 2020) and that f_esc_ partially captures the response of LUE to diffuse light and therefore both parameters have a temporal correlation (Kim et al., 2021), normalizing ${\mathrm{SIF}}_{760}^{\mathrm{leaf}}$ with PAR or ${\mathrm{APAR}}_{\mathrm{chl}}$ is a well-accepted approach to relate larger scale SIF measurements to the mechanistic regulation of photosynthesis, which is normally parameterized on the leaf level. The now accessible relationship between available light energy and the emission efficiency shows a diurnal dynamic dependent on other photon pathways, including NPQ and photosynthetic activity (Pinto et al., 2016; Porcar-Castell et al., 2014; van der Tol et al., 2009).

The $\varepsilon_{\mathrm{SIF}\left(\mathrm{PAR}\right)}$ and $\varepsilon_{\mathrm{SIF}\left(\mathrm{FCVI}\right)}$ hysteresis patterns of winter wheat (Figs. 8b and e) were very similar to a hysteresis for corn (Pinto et al., 2016). Both winter wheat hysteresis patterns can be mechanistically explained as follows: The first positive relationship in the morning, already observed many times, indicates an increase in photochemical activity with an associated increase of the emission efficiency under increasing light availability (Campbell et al., 2019). This relationship becomes saturated around noon, when light intensities reach and exceed the maximum photosynthetic electron transport rates. If external conditions, such as temperature, vapor pressure deficit, and water availability become stressful, which most often occurs in the early afternoon, stomata close and internal leaf CO_2_ availability may be limited. Under these conditions, the capacity of linear photosynthetic electron transport often exceeds its capacity and non-photochemical energy dissipation mechanisms (NPQ) are upregulated to dissipate excessive energy in the photosynthetic apparatus. This quenching causes a reduction of the emission efficiency of fluorescence, as reported by Amoros-Lopez et al. (2008), which is also visible in the diurnal trend of $\varepsilon_{\mathrm{SIF}\left(\mathrm{FCVI}\right)}$ of the investigated wheat fields presented in Fig. 3e. During the afternoon, the positive correlation between $\varepsilon_{\mathrm{SIF}\left(\mathrm{PAR}\right)}$ and PAR, as well as $\varepsilon_{\mathrm{SIF}\left(\mathrm{FCVI}\right)}$ and ${\mathrm{APAR}}_{\mathrm{chl}}$ (Fig. 8b and e), recovered, but at a lower level because of still active NPQ mechanisms, which have a longer half-time for their downregulation (Kromdijk et al., 2016; van der Tol et al., 2009).

The sugar beet and the fruit tree hysteresis based on $\varepsilon_{\mathrm{SIF}\left(\mathrm{PAR}\right)}$ were similar to those of winter wheat (Figs. 8a and c). In contrast, the hysteresis of both crops distinctly changed for $\varepsilon_{\mathrm{SIF}\left(\mathrm{FCVI}\right)}$ (Figs. 8d and f). For sugar beet, this was related to the lower absorption of PAR by leaf chlorophyll in the afternoon compared to the morning (Figs. 3c and S3), possibly caused by the changing leaf angularity throughout the day. Additionally, the observed $\varepsilon_{\mathrm{SIF}\left(\mathrm{FCVI}\right)}$ hysteresis could be an indicator that sugar beet did not show any signs of stress on this day. Sugar beet is generally well-adapted to the climatic conditions and the large root provides sufficient water storage to usually prevent stomatal closure under normal summer conditions. Thus, we assume that NPQ mechanisms were not excessively upregulated in sugar beet on that day and therefore no clear hysteresis was detected. This is in accordance with results achieved by Cerovic et al. (1996), who presented a similarly shaped hysteresis based on active fluorometric measurements of non-stressed sugar beet leaves. Additionally, Cerovic et al. (1996) showed how the hysteresis shape changed when sugar beet leaves were exposed to water stress. It would be interesting if the same hysteresis shape could also be derived from HyPlant data covering a sugar beet field suffering from water shortage. In Fig. 3e, in which $\varepsilon_{\mathrm{SIF}\left(\mathrm{FCVI}\right)}$ of sugar beet is additionally plotted as a function of time, a reduction of the emission efficiency in the early afternoon is also not visible. This is another indicator that the plants were not stressed and therefore NPQ mechanisms were not excessively upregulated.

The $\varepsilon_{\mathrm{SIF}\left(\mathrm{FCVI}\right)}$ hysteresis of winter wheat suggested different physiological conditions. Winter wheat has no drought avoidance mechanisms and it can be assumed that photosynthetic electron transport became saturated and stomatal closure occurred in combination with an upregulation of NPQ mechanisms. This is clearly visible in the saturated $\varepsilon_{\mathrm{SIF}\left(\mathrm{FCVI}\right)}$ values around solar noon and the greatly reduced afternoon values (Fig. 3e and 8e), which point towards still upregulated NPQ mechanisms.

In a study of van der Tol et al. (2014) a reduction of the SIF emission efficiency at leaf level from the morning to noon has been reported. Such a decrease of $\varepsilon_{\mathrm{SIF}\left(\mathrm{FCVI}\right)}$ was not observed for sugar beet an winter wheat in this study (Fig. 3e). One reason is that the intensity of PAR/APAR_chl_ was already at a high level during the acquisition of the first airborne data set at 10:10, and thus a potential early morning decrease could not be covered with the used airborne data. Furthermore, van der Tol et al. (2014) used an active fluometric device to collect measurements at leaf scale. The emission efficiency derived from active measurements, however, is not completely comparable to spectroscopy-based measurements of SIF emission efficiency, which could be another reason why the presented results deviate from those of van der Tol et al. (2014).

The $\varepsilon_{\mathrm{SIF}\left(\mathrm{FCVI}\right)}$ hysteresis of the fruit trees do not allow for a physiological interpretation of the quenching mechanisms (Fig. 8f). It is assumed that the applied downscaling procedure could not completely account for all structural effects of this geometrically complex canopy type. The investigated fruit tree parcels, which consisted of different species and trees of different ages and structures planted in clear rows, most likely introduced various shortcomings in the applied downscaling approach and thus a detailed physiological interpretation of the results would not be meaningful. Kernel-driven methods, as presented by Hao et al. (2021), could be used as alternative approaches to determine f_esc_ of fruit trees more precisely, since it was demonstrated that they better account for the complexity of three-dimensional canopies planted in rows.

### 4.3 Reliability and limitations of the study

The presented airborne data set is unique and for the first time allowed the investigation of the spatial and temporal dynamics of far-red SIF at canopy and leaf level over the course of a day. Nevertheless, it is important to discuss the reliability and limitations of some aspects of this study.

Three contrasting crop types were investigated to study the influence of their canopy structure on the SIF emission at canopy scale. The obtained results for the two structurally simple but still contrasting crops, sugar beet and winter wheat, are promising and clearly demonstrate that different canopy geometries lead to different f_esc_.

The FCVI was developed to quantify the combined effect of fAPAR and $f_{\mathrm{esc}}$ and thus to separate the physiologically related variation in far-red SIF from the non-physiologically related variation (Yang et al., 2020). In this context, especially for plants characterized by a changing leaf geometry over the course of the day, e.g., sugar beet, the results clearly demonstrate that calculating $\varepsilon_{\mathrm{SIF}}$ based on the FCVI ($\varepsilon_{\mathrm{SIF}\left(\mathrm{FCVI}\right)}$) that considers APAR_chl_ (Eqs. (8), (9), (10)) instead of PAR can be regarded as more useful as a means of detecting variations in plant physiology. This is because only radiation absorbed by chloroplasts can be emitted as far-red SIF. The advantage of $\varepsilon_{\mathrm{SIF}\left(\mathrm{FCVI}\right)}$ is that it can be determined without knowledge of APAR_chl_ and $f_{\mathrm{esc}}$ (Eq. 10). However, if APAR_chl_ is known, the FCVI can also be used to calculate $f_{\mathrm{esc}}$. An empirical relationship based on SCOPE simulations between ${\mathrm{fAPAR}}_{\mathrm{green}}$ and the WDRVI (Liu et al., 2019), in combination with a factor k also estimated from SCOPE (Du et al., 2017), formed a basis for deriving fAPAR_chl_ from HyPlant DUAL TOC reflectance data in this study. Since SCOPE does not account for leaf clumping effects, applying this method only allowed for an approximation of fAPAR_chl_. Furthermore, Liu et al. (2019) generated a look-up table with SCOPE, which covered a wide range of LAI, LCC and leaf inclination angles, which possibly dominate sun zenith angle effects on the relationship between fAPAR_green_ and WDRVI in the course of a day. Fig. S4 shows the relationships for the investigated crops based on SCOPE simulations using the average LAI and LCC derived from the PROSAIL inversion results of each crop (Fig. S2). All parameters were kept constant in the simulation process except the sun zenith angle (varied for all crops), sun azimuth angle (varied for all crops) and the leaf inclination distribution function parameter a (LIDFa) (only varied for sugar beet). Table S5 summarizes the used SCOPE input parameters. For winter wheat and fruit trees characterized by a spherical and constant leaf angle distribution the proposed equation by Liu et al. (2019) (Eq. 2) enabled a precise estimation of fAPAR_green_. In contrast, only a moderate relationship was found for sugar beet, which could be related to the changing leaf orientation from predominantly erectophile in the morning to predominantly planophile in the afternoon. For these reasons, future studies should include in situ measurements of APAR_green_ or APAR_chl_ to enable a sound validation of this important parameter derived from remote sensing data. An over- or underestimation of fAPAR_chl_ in this study could have introduced possible uncertainties in the determination of $f_{\mathrm{esc}}$ and thus also in the subsequent estimation of SIF_760_ at leaf scale.

The FCVI is not suitable for sparse canopies with a reflecting soil background and therefore Yang et al. (2020) recommend excluding observations with a FCVI lower than 0.18. In the present study, a low number of pixels recorded within the fruit tree parcels had FCVI values slightly lower than 0.18 (Fig. 3b). Besides the structural complexity, this could be a reason why the downscaling results of the fruit trees remained inconclusive and thus it is not recommended to simply use the presented downscaling approach for geometrically complex tree ecosystems. NIRv (Zeng et al., 2019) and kernel-driven methods (Hao et al., 2021) could be alternative approaches to better account for soil background effects and a more complex canopy geometry. However, further research is needed to verify this.

In future studies, diurnal SIF (e.g. from FLUOWAT) and active fluorescence measurements (e.g. from pulse-amplitude modulation (PAM)) of leaves collected in parallel to the airborne data acquisition could provide important additional information to directly prove the accuracy of f_esc_ and ${\mathrm{SIF}}_{760}^{\mathrm{leaf}}$ estimated from HyPlant data. Furthermore, the proposed approach needs to be tested for more vegetation types and phenological stages, including conditions in which plants are exposed to different kinds of stress. It would also be interesting to investigate additional data sets recorded at different times during the growing season. This may lead to a better understanding of the changing influence of the canopy structure (represented by f_esc_) on ${\mathrm{SIF}}_{760}^{\mathrm{canopy}}$ over the season. Studies providing initial insights into seasonal trends of f_esc_ of different crops derived from point spectrometer measurements mounted on towers at different sites (Dechant et al., 2020; Liu et al., 2020) and derived from OCO-2 and TROPOMI satellite data (Wang et al., 2020) have already been published.

## 5 Conclusion

Unraveling the structural and physiological contributions that determine apparent SIF retrieved from remote sensing data is essential to avoiding the misinterpretation of such SIF retrievals and exploiting the full information content inherent to this new and complementary remote sensing signal. There is growing evidence from literature and the results of the present study that structural sensitivities in remotely sensed SIF can be successfully compensated for with complementary information inherent to acquired spectral data.

Taking the results of our spatio-temporal assessment into account, we conclude that the FCVI has great potential to approximate structural interferences (expressed as $f_{\mathrm{esc}}$) in order to eventually scale SIF_760_ of sugar beet and winter wheat from canopy to leaf level. We conclude that it is sufficient to obtain the required scaling factors once a day for homogeneous canopies (i.e., cereal crops) to reliably scale SIF_760_ from canopy to leaf level. However, it is strongly recommend that instantaneous $f_{\mathrm{esc}}$ is calculated with respect to the diurnal period of interest for more complex canopies (i.e., sugar beet).

Our unique field study enabled the assessment of the spatial and temporal dynamics of structural interference and the relation between leaf- and canopy-level SIF_760_ dynamics. We suggest that such experiments should be expanded to include an even wider range of vegetation types, covering large structural gradients and also across seasons, so as to include a wider range of growth stages. We also recommend complementing spectroscopy-based SIF estimates with active fluorescence techniques such as PAM or light-induced fluorescence transient (LIFT) measurements (Murchie et al., 2018), or gas exchange measurements, to acquire quantitative information on the degree of NPQ mechanisms, stomatal opening, and functional stress responses. Combining the results of this study, a methodology for the downscaling of SIF from canopy to leaf scale was successfully applied to a diurnal data set of high-resolution airborne image data. Although we are confident that our downscaling approach can also be applied successfully to spatial lower resolution airborne and satellite image data, further research is needed to confirm this assumption.

Pending further assessments that consider an even wider range of plant types, our approach can be considered as a new strategy to compensate for the effects of canopy structure and to isolate the physiological contribution inherent to canopy-scale SIF. The far-red SIF estimates obtained at leaf scale are particularly interesting when environmental constraints limit photosynthetic processes and when ecosystem models require a better physiology-based parameterization.

## Declaration of Competing Interest

The authors declare that they have no known competing financial interests or personal relationships that could have appeared to influence the work reported in this paper.

## Appendix A Supplementary data

Supplementary material Image 1
