# Supplementary material for: Downscaling of far-red solar-induced chlorophyll fluorescence of different crops from canopy to leaf level using a diurnal data set acquired by the airborne imaging spectrometer HyPlant
Source: Remote Sens Environ. 2021 Oct;264:112609. doi: 10.1016/j.rse.2021.112609 (PMC8447579; doi:10.1016/j.rse.2021.112609)
Supplement: Supplementary file 1 — Supplementary material [file mmc1.pdf]

Supplemental Material

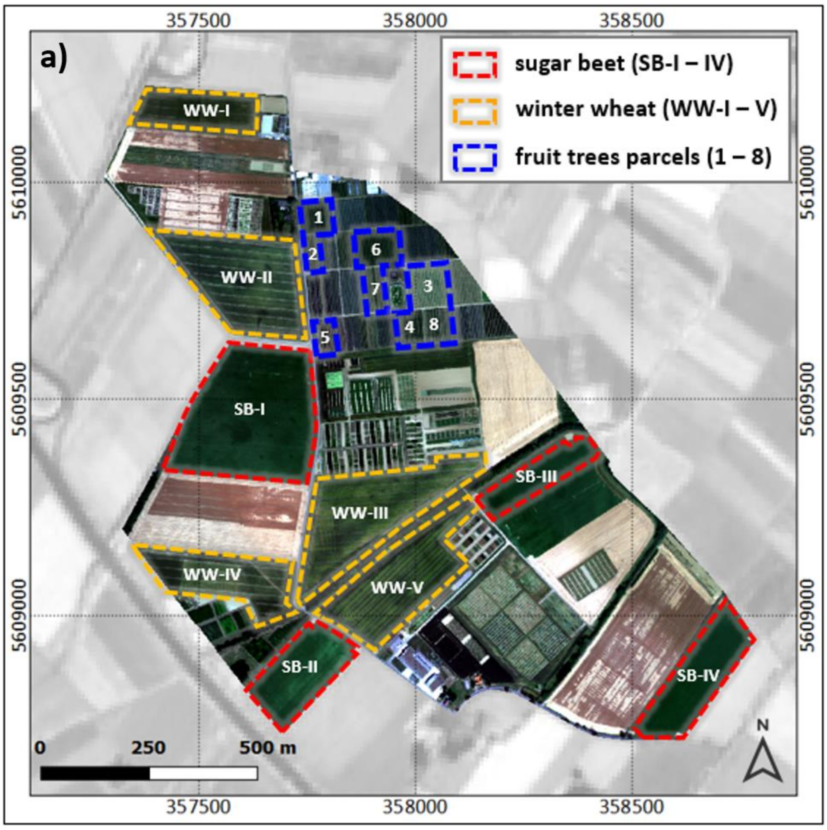

b)

Field	Variety	Size (ha)	Days after sowing*
SB-I	Lisanna	8.83	79-82
SB-II	Alcedo	3.68	80-83
SB-III	BTS 440	2.63	80-83
SB-IV	Alcedo	3.65	81-84
WW-I	Lomerit	2.99	252-255
WW-II	Reform	5.94	260-263
WW-III	Reform	6.11	257-260
WW-IV	Rubisko	4.11	231-234
WW-V	Reform	5.05	246-249

* with regard to HyPlant data acquisition (26.-29.06.18)

c)

Parcel	Species	Size (ha)	Age (years)
1	Apple	0.50	4
2	Apple	0.28	0.5
3	Apple	0.98	0.5
4	Apple	0.58	3
5	Apple	0.38	2
6	Pear	1.18	4
7	Pear	0.60	1
8	Pear	0.48	1

Fig. S1: Complementary information about the investigated sugar beet and winter wheat fields as well as fruit tree parcels. (a) HyPlant DUAL top-of-canopy (TOC) true-color composite (RGB 640/550/460 nm) of Campus Klein-Altendorf acquired on June 29th 2018 with dashed lines highlighting the locations of the sugar beet (red) and winter wheat fields (orange) as well as the fruit tree parcels (blue). Background: Sentinel-2 (Band 8) from June 27th 2018. (b) Investigated sugar beet and winter wheat fields with information about the variety, size and days after sowing. (c) Investigated fruit tree parcels with information about species, size and tree age.

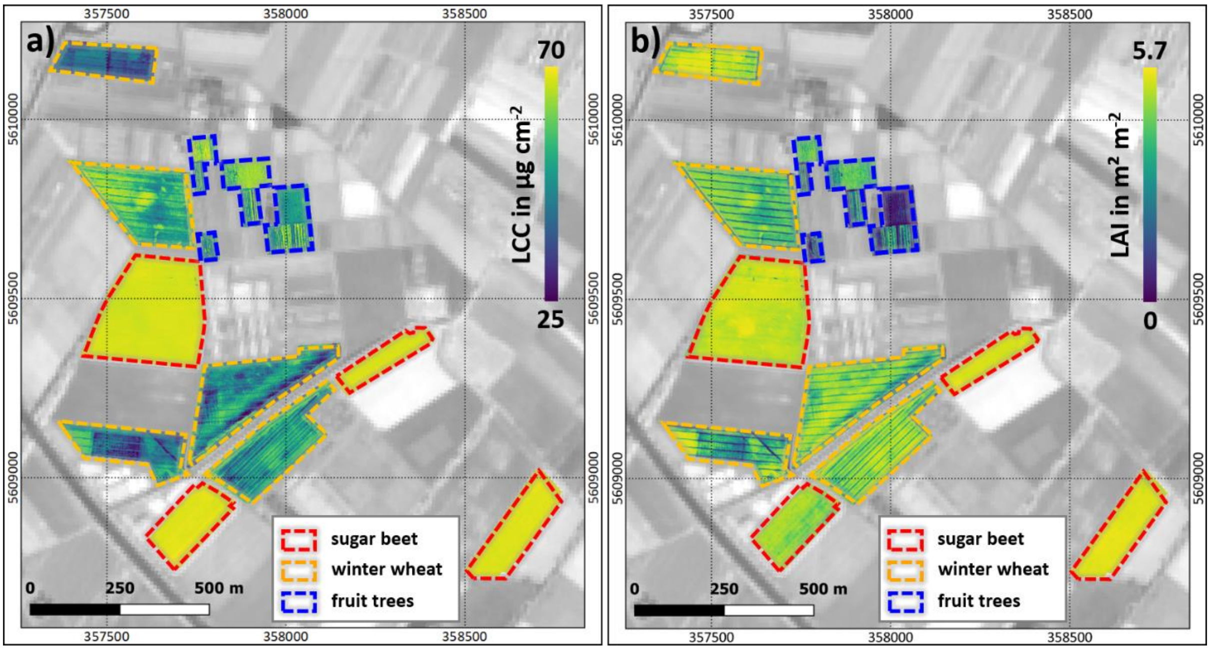

Fig. S2: Derived leaf chlorophyll content (LCC) (a) and leaf area index (LAI) map (b) based on a PROSAIL inversion of the HyPlant DUAL data set recorded during the third overflight at 12:30 CEST on June 29th 2018 with dashed lines highlighting the locations of the sugar beet (red) and winter wheat fields (orange) as well as the fruit tree parcels (blue). Background: Sentinel-2 (Band 8) from June 27th 2018.

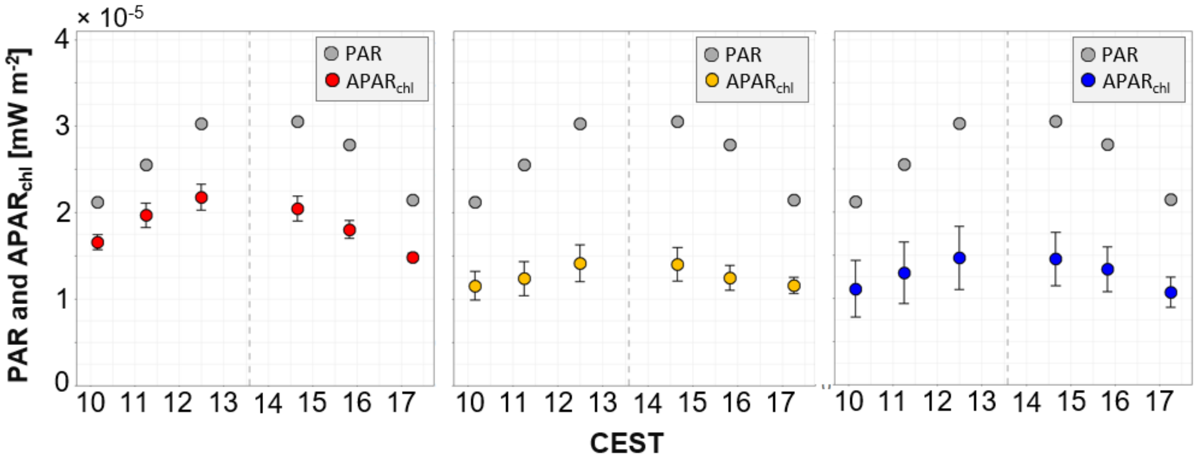

Fig. S3: Diurnal dynamic of incoming photosynthetically active radiation (PAR) and the fraction of PAR absorbed by leaf chlorophyll ($APAR_{chl}$) of sugar beet (red), winter wheat (orange) and fruit trees (blue). The colored circles represent averaged $APAR_{chl}$ values of the observed crops with corresponding standard deviations, while the grey circles indicate PAR measured at the weather station during the time of the HyPlant overflights. The vertical dashed grey lines show the time of local solar noon.

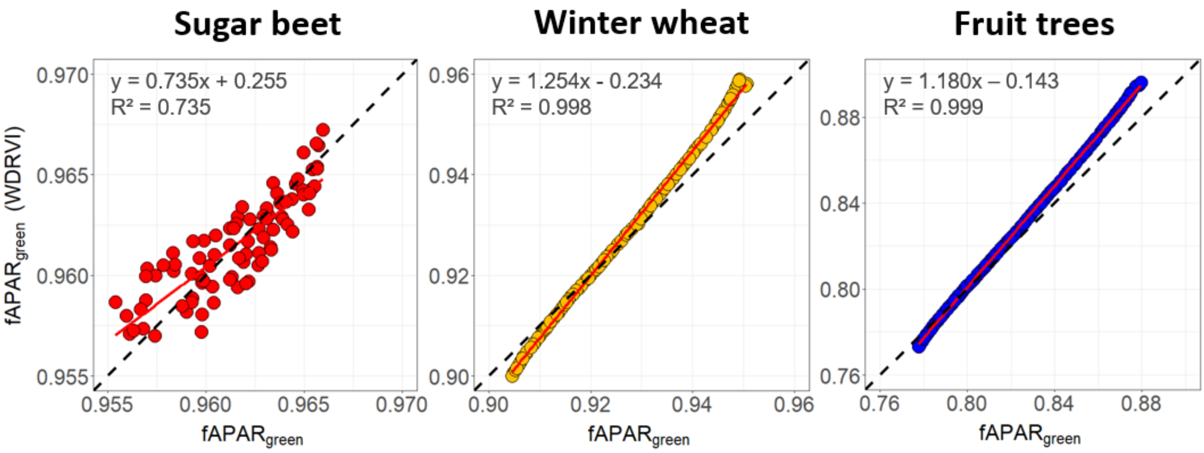

Fig. S4: SCOPE simulations showing the relationship of $fAPAR_{green}$ and $fAPAR_{green}$ estimated with the WDRVI using Equation 4 as proposed in Liu et al. (2019). The solid red line represents the regression line, while the dashed black line represents the 1:1 line.

Tab S1: Correlation coefficients calculated for corresponding pixels of the canopy (SIF_{760}^{canopy}) and leaf SIF_{760} (SIF_{760}^{leaf}) maps derived for the three investigated crops sugar beet, winter wheat and fruit trees from the six HyPlant overflights.

Day	Time	Sugar Beet (SB)	Winter Wheat (WW)	Fruit Trees (FT)
27 June 2018	10:10	0.923	0.946	0.954
29 June 2018	11:15	0.897	0.953	0.873
29 June 2018	12:30	0.941	0.966	0.914
29 June 2018	14:40	0.947	0.958	0.828
29 June 2018	15:50	0.949	0.943	0.810
26 June 2018	17:15	0.914	0.931	0.960

Tab S2: Correlation coefficients calculated for corresponding pixels of the canopy (SIF_{760}^{canopy}) and leaf SIF_{760} (SIF_{760}^{leaf}) maps derived for the individual sugar beet fields (SB-I – SB-IV) from the six HyPlant overflights.

Day	Time	SB-I	SB-II	SB-III	SB-IV
27 June 2018	10:10	0.961	0.950	0.982	0.972
29 June 2018	11:15	0.849	0.951	0.906	0.964
29 June 2018	12:30	0.890	0.910	0.962	0.955
29 June 2018	14:40	0.890	0.703	0.751	0.897
29 June 2018	15:50	0.827	0.908	0.810	0.921
26 June 2018	17:15	0.829	0.994	0.975	0.954

Tab S3: Correlation coefficients calculated for corresponding pixels of the canopy (SIF_{760}^{canopy}) and leaf SIF_{760} (SIF_{760}^{leaf}) maps derived for the individual winter wheat fields (WW-I – WW-V) from the six HyPlant overflights.

Day	Time	WW-I	WW-II	WW-III	WW-IV	WW-5
27 June 2018	10:10	0.988	0.973	0.965	0.963	0.982
29 June 2018	11:15	0.988	0.949	0.959	0.957	0.938
29 June 2018	12:30	0.987	0.971	0.983	0.990	0.921
29 June 2018	14:40	0.949	0.939	0.979	0.993	0.939
29 June 2018	15:50	0.944	0.986	0.992	0.997	0.979
26 June 2018	17:15	0.957	0.985	0.989	0.996	0.927

Tab S4: Correlation coefficients calculated for corresponding pixels of the canopy (SIF_{760}^{canopy}) and leaf SIF_{760} (SIF_{760}^{leaf}) maps derived for the individual fruit tree parcels (FT-1 – FT-8) from the six HyPlant overflights.

Day	Time	FT-1	FT-2	FT-3	FT-4	FT-5	FT-6	FT-7	FT-8
27 June 2018	10:10	0.889	0.942	0.999	0.919	0.911	0.931	0.952	0.959
29 June 2018	11:15	0.782	0.812	0.992	0.789	0.898	0.568	0.856	0.922
29 June 2018	12:30	0.898	0.950	0.992	0.903	0.912	0.858	0.939	0.969
29 June 2018	14:40	0.867	0.773	0.766	0.864	0.914	0.670	0.867	0.954
29 June 2018	15:50	0.677	0.904	0.702	0.877	0.916	0.802	0.911	0.926
26 June 2018	17:15	0.859	0.929	0.922	0.947	0.896	0.962	0.970	0.977

Tab S5: Input parameters used for the SCOPE simulations to verify the relationship between $fAPAR_{green}$ *and WDRVI proposed by Liu et al. (2019). LCC = leaf chlorophyll content, LAI = leaf area index, SZA= sun zenith angle, SAA = sun azimuth angle, LIDFa = leaf inclination distribution function parameter a, LIDFb = leaf inclination distribution parameter parameter b.*

Scope parameters	Sugar beet	Winter wheat	Fruit trees
LCC in µg cm^{-2}	67.63	48.57	54.64
LAI	5.21	4.21	2.41
SZA in °	20–60	20–60	20–60
SAA in °	0–180	0–180	0–180
LIDFa	-0.35–0.35	-0.35	-0.35
LIDFb	0	-0.15	-0.15
